# The Pharmacodynamics of the p53-Mdm2 Targeting Drug Nutlin: The Role of Gene-Switching Noise

**DOI:** 10.1371/journal.pcbi.1003991

**Published:** 2014-12-11

**Authors:** Krzysztof Puszynski, Alberto Gandolfi, Alberto d'Onofrio

**Affiliations:** 1 Silesian University of Technology, Institute of Automatic Control, Gliwice, Poland; 2 Istituto di Analisi dei Sistemi ed Informatica "A. Ruberti" - CNR, Rome, Italy; 3 Department of Experimental Oncology, European Institute of Oncology, Milan, Italy; 4 International Prevention Research Institute, Lyon, France; National Research Council of Canada, Canada

## Abstract

In this work we investigate, by means of a computational stochastic model, how tumor cells with wild-type p53 gene respond to the drug Nutlin, an agent that interferes with the Mdm2-mediated p53 regulation. In particular, we show how the stochastic gene-switching controlled by p53 can explain experimental dose-response curves, i.e., the observed inter-cell variability of the cell viability under Nutlin action. The proposed model describes in some detail the regulation network of p53, including the negative feedback loop mediated by Mdm2 and the positive loop mediated by PTEN, as well as the reversible inhibition of Mdm2 caused by Nutlin binding. The fate of the individual cell is assumed to be decided by the rising of nuclear-phosphorylated p53 over a certain threshold. We also performed *in silico* experiments to evaluate the dose-response curve after a single drug dose delivered in mice, or after its fractionated administration. Our results suggest that dose-splitting may be ineffective at low doses and effective at high doses. This complex behavior can be due to the interplay among the existence of a threshold on the p53 level for its cell activity, the nonlinearity of the relationship between the bolus dose and the peak of active p53, and the relatively fast elimination of the drug.

## Author Summary

P53 is an antitumor gene regulating vital cellular functions such as repair of DNA damage, cellular suicide, and cell proliferation: in many tumors p53 is lowly expressed and/or mutated. Drugs targeting the biomolecular network of p53 are becoming important. The network includes the key proteins Mdm2 and PTEN, whose production is regulated by p53, and which, in turn, enact positive and negative feedbacks on p53. Drug Nutlin, inhibiting the p53 inhibitor Mdm2, might be important for tumors where p53 is underproduced but unmutated. We investigate the cellular mechanism of action of Nutlin. The basic concept of our mathematical model is that the experimentally observed cell-to-cell variability of Nutlin efficacy is caused by the randomness of gene activation/deactivation of Mdmd2 and PTEN. Indeed, the abundance/scarceness of p53 regulates the probability that the relative genes are active or inactive. The model reproduced the experimental cell-specific response to different doses of Nutlin (dose-response curves) in some types of tumor cells. Much clinical research focus on 'metronomic' drug delivery regimens, where instead of giving large doses with long intervals, smaller doses are frequently delivered. In our simulations, dose-splitting of Nutlin produced a response generally worse than the response to a single dose.

## Introduction

The p53 gene is an important oncosuppressor gene, and its product is heavily involved in the control of both cell proliferation and cell differentiation. It is well known that p53 triggers cell cycle arrest and even apoptotic pathways in response to moderate and, respectively, strong stress signals [1]. Concerning cell differentiation, p53 suppression induces a strong increase of the probability of symmetric division in breast stem cells [2], and drug-driven activation of p53 induces rapid differentiation of human embrionic stem cells [3].

Underexpression of p53 is often observed in tumors carrying wild-type p53 [1], [4]. This phenomenon is caused by overexpression of Mdm2 protein, the main competitor of p53 [1], [4]. For example, this may occur when the gene p14, which inhibits Mdm2 by sequestering it into the nucleus, is deleted, as observed in breast, brain and lung cancers [1]. Another cause leading to the underexpression of wild-type p53 is given by Mdm2 amplification [1], [4]. Moreover, binding to viral proteins in infected cells causes underexpression of p53 in chronic infection-related cancers [1], [4]. Underexpressed wild-type p53 is seen as a primary candidate target for antitumor therapies based on chemical molecules [1] or siRNA [5].

Among the p53-targeting drugs, a prominent role is played by Nutlins [6], a family of small molecules able to bind Mdm2 exactly in the "binding pockets" where p53 binds, so impeding the formation of p53-Mdm2 complexes and inducing a rapid p53 level increase. Since the activation of p53 may cause the triggering of both the apoptotic pathways and the differentiation of tumor stem cells, Nutlins are regarded as potentially important antitumoral agents. In their study, Vassilev et al. [6] showed a potent antitumor activity of Nutlins on wild-type p53 tumor cell lines, such as HCT116, RKO and SJSA-1 cells, whereas only a marginal effect on mutant p53 cell lines (such as SW 480, MDA-MB-435, PC3) was observed. The same research group [7] later found that different Nutlins subtypes may have a differential action on different tumor cell lines. A number of other preclinical studies reported that Nutlin is an effective antitumor drug for important types of cancers carrying dysfunctional wild-type p53. The antineoplastic action of Nutlin on chronic B-cell lymphocytic leukemia with wild-type p53 has been shown [8], documenting a series of synergies with doxorubicin. Nutlin is active against prostate cancer cells retaining wild-type p53 and androgen receptor signaling [9], and works by inhibiting their proliferation via cell cycle arrest and apoptosis. Nutlin-3a is also active in Hodgkin lymphoma, where p53 is rarely mutated [9]. In Ewing's sarcoma cells, Nutlin-3 restores wild-type p53 functions, with cancer growth inhibition and apoptosis induction, whereas no effect was observed for cells with mutated p53 (the mutation, however, affects only 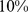 of those tumors). In addition, Nutlin is active against human glioblastoma multiforme [10], where 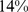 of patients carry amplifications of Mdm2. In this case, Nutlin was active in the wild-type p53 glioblastomas, where it also caused cell senescence. In [10], it has been explicitly noticed that cell lines can significantly differ in their apoptotic response to similar levels of p53 activation. We may observe that the Nutlin-mediated restoration of p53 levels does not automatically guarantee beneficial effects if other modules of p53-related pathway are dysfunctional. For example, Ma et al. [11] showed that Nutlin-3 is unable to induce p53-related apoptosis in cells where p53-Ser46 phosphorylation is defective. In retinoblastoma p53 is intact, but it is silenced by MDMX overexpression [12], [13]. A preclinical study [13] has reported strong activity of locally-administered Nutlin-3a against retinoblastoma, and synergy with topotecan. Recently, it has been shown that Nutlin overcomes resistance to Vemurafenib in melanoma lines [14], and to Cisplatin in ovarian cancer cells [15]. Finally, in both the above-mentioned studies concerning the role of p53 in the differentiation of stem cells [2], [3], Nutlin was the drug used for p53 activation.

The above experimental findings on the effect of Nutlin on wild-type p53 tumors can be roughly summarized as follows: the binding of Nutlin to Mdm2, by inactivating the main antagonist of p53, leads to increasing the p53 level, which negatively influences the tumor growth, in part because of the onset of cell arrest and apoptosis, in part – for stem cell-based tumors – by establishing in cancer stem cells a more physiological pathway of asymmetric cell division. However, the design and implementation of efficacious therapies requires going beyond a mere descriptive approach, which disregards the kinetics and the quantitative features of the phenomena. Valid tools can be provided by Systems Biology, which is able to integrate information from multiple sources in a coherent quantitative model by using mathematics and bioinformatics [16].

Indeed, the role of p53 has elicited the interest of many computational biologists since the seminal experimental/modeling work conducted by Alon et al. [17], where the onset of oscillations in p53 concentration during the response to radiation stress was shown. A major contribute was given by Ciliberto et al. [18], who stressed the role of both negative and positive feedbacks in p53/Mdm2 interplay. Other authors [19], [20], [21], [22] stressed the role of delays in the p53/Mdm2 network, although recently it has been noticed [23], [24], [25] that the use of explicit delays to explain p53 oscillations (and other dynamical features) may be avoided by including in the model the complexes formed by p53 and Mdm2. Sturrock et al. [26], [27] and Dimitrio et al. [28] proposed models where the intra-cellular spatial diffusion of p53 is represented and used as causative agent for the onset of the observed oscillations. Laise et al. [29] recently proposed a model of the hypoxia-related apoptotic pathway p53/HIF-1/p300 networks.

Apart from [29], all the above-mentioned mathematical models have focused only on the p53/Mdm2 network. However, PTEN protein plays a major role in p53 regulation [30], [31] and should not be neglected. A stochastic model of p53/Mdm2/PTEN interplay during environmental stresses was proposed by Puszynski et al. [32], and that model forms the basis of our work. Zhang and colleagues in [33] included the p53/PTEN/Akt/Mdm2 positive feedback in their deterministic model, although not directly including PTEN among the state variables, showing its relevance in the interplay between p53/Mdm2/PTEN network and p21 network. Moreover, in [34] they more directly analyzed the role of the delicate trade-off between the p53/PTEN/Akt/Mdm2 and the ATM/p53/Wip1 feedbacks during the process of DNA damage response.

The present work is aimed at building – and, to some extent, comparing with data – a quantitative model of Nutlin pharmacodynamics at the single-cell level that explicitly includes the main biochemical network regulating p53, along with the transcriptional feedbacks. These chemical reactions, mostly following the mass-action law, are converted into a hybrid stochastic model, i.e., a model including both differential equations and birth-and-death stochastic processes.

The model, besides Mdm2, includes PTEN and ubiquitins as two other major players shaping the dynamics of the p53 network. Indeed p53 is a transcription factor for PTEN, which in turn (through PIP and Akt) induces Mdm2 phosphorylation by means of a positive feedback [30], [31]. Since these processes are enacted with timescales not substantially different to those typical of Mdm2-p53 interactions, PTEN cannot be eliminated from the model via a quasi-steady state approach. On the contrary, since we are interested in analyzing the dynamics of cell response to time-varying Nutlin concentration, PTEN is a primary actor and its subnetwork has to be explicitly represented. The role of p53 ubiquitination in the dynamics of p53 has been emphasized in [18]. Moreover, we trace p53-Mdm2 complexes, as suggested in [23], [24]. Concerning the pharmacodynamics of Nutlin, which is the key issue of our study, the competition of Nutlin with p53 for Mdm2 binding is coupled in our model with a simple linear cell uptake. We show by simulations that the stochasticity of gene-switching may account for the observed inter-cell variability of the response. Moreover, our simulations suggest that dose-splitting could reduce the anti-tumoral effect of Nutlin *in vivo*. Considerations on the limits of the model, the clinical applicability of the drug, and the future research direction conclude this work.

## Models

We propose a model of the Nutlin pharmacodynamics based on the detailed stochastic model of the p53/Mdm2/PTEN network presented in [32]. Here, we specifically add to that model: i) an equation for the Nutlin concentration into the target cell; ii) the description of the competition of Nutlin with p53 in binding Mdm2; iii) the description of the mono- and bi-ubiquitination of p53, with the definition of an effective degradation rate of the bi-uquitinated molecule to account for the fast degradation of poly-ubiquitinated forms.

**Figure 1:**
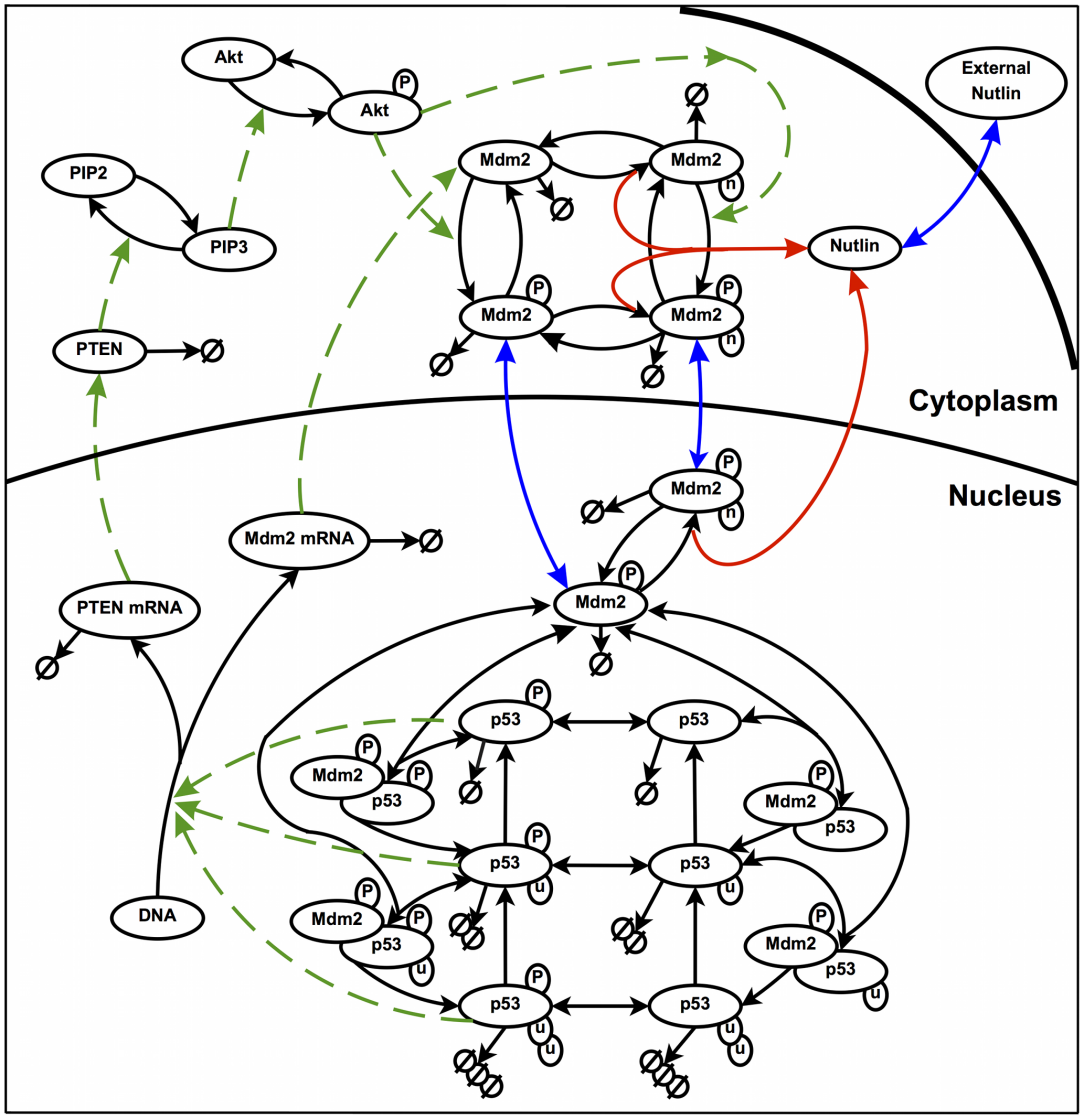
Pictorial view of the p53-Mdm2-PTEN interactions in the presence of Nutlin. Black solid arrows: chemical reactions; red solid arrows: chemical reactions involving Nutlin; green dashed arrows: induction of activities; blue solid arrows: translocations. P, phosphate group; u, ubiquitin; n, Nutlin; the symbol 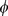 denotes degradation.

Roughly speaking, the model of p53 regulation in the absence of Nutlin relies on two feedback loops: a negative loop, coupling p53 with its immediate down-regulator Mdm2, and a positive loop, which involves PTEN, PIP3 and Akt. The existence of the negative feedback assures homeostasis of healthy cells and oscillatory responses of DNA-damaged cells. The positive feedback loop compensate the negative coupling between Mdm2 and p53 by sequestering Mdm2 in cytoplasm. Later on in this section, we will give a more detailed description of these feedbacks. In the model, we distinguish between three physical compartments: the nucleus, the cytoplasm and the extra-cellular space, where, however, only Nutlin is present. In Fig. 1, a diagrammatic representation of the reactions is shown. To mathematically model our complex network, a first possibility is to explicitly model all the reactions as birth-and-death stochastic processes, using the Gillespie algorithm [35] for their simulation. This algorithm is exact for mass action law-based models but it may often be extremely time consuming, even for medium-small networks. This may happen in the presence of two or more largely different timescales, a ubiquitous phenomenon in biology [36]. Here, similarly to the p53/Mdm2/PTEN model under genetic stress proposed in [32], we adopt a hybrid approach, where the process of gene activation-deactivation is stochastic and it is simulated by means of the Gillespie algorithm, whereas the other "fast" reactions are modeled by means of ODEs mostly built on the basis of mass action law. The complete list of model variables is given in S1 Text.

Notice that in our model, variable subscripts have the following meaning: 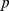 - phosphorylated, 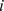 - inactive (by binding of Nutlin molecule), 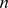 - nuclear (a variable without the 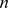 subscript means a cytoplasmic quantity).

### Gene-switching stochasticity

As in [32], we follow the experimental evidence [37], [38], [39] that transcription factors regulate the probability that a given gene is ON or OFF, rather than the mRNA transcription rate. The ON/OFF *random gene-switching* results in a burst production of mRNA molecules, and introduces (also in the idealized case of the absence of downstream sources of randomness) a large level of stochasticity in the dynamics of cell regulation networks [40], [41], [42], [43]. Denoting by 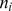 the number of gene copies of a generic gene 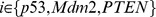, the number of copies of gene 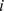 active at time 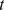 is 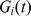 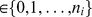. We assume that the deactivation rate of the single gene copy is constant, and that the deactivation events are independent, so that:
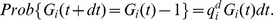



As far as the activation rates are concerned, we recall that the p53 protein is a transcription factor for both Mdm2 and PTEN [44], and that p53 phosphorylation enhances p53 activity in transcription [45]. Moreover, p53 is involved in co-translational dimerization and in post-translational dimerization of dimers [46]. Although such tetramerization appears to be rather inefficient in solution, p53 dimers exhibit high cooperativity in DNA binding, with a Hill coefficient of 1.8 [47], and mutated p53 with impaired tetramerization binds DNA with an affinity six-fold less than the affinity of the wild-type protein. Thus, we assume that p53 in the cell is mainly present as a dimer, and that its activity in transcription requires tetramerization at the level of DNA binding. These assumptions yield to
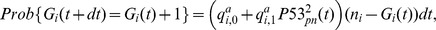
where 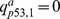.

We assume that when a gene copy is active, transcription proceeds at a constant rate. We remark that although all the other chemical reactions of the model are described by ordinary differential equations, the time-courses of *all* the chemical species will be actually given by *stochastic processes* since all the reactions are ultimately driven by gene activation. Finally, note that the random fluctuations of the gene activation may be seen as a bounded stochastic process [48] perturbing the system constituted by proteins and transcripts, some of which, in turn, feedback on the dynamics of this peculiar kind of noise.

### The feedback loops

#### Negative feedback loop: p53-Mdm2 binding and Mdm2-mediated polyubiquitination of p53

The subcellular localization of p53 depends on the cell cycle phase. Because p53 is predominantly nuclear in the G0 and G1 phase, and in the remaining phases is rapidly translocated to the nucleus in response to stress [49], we assume that the dynamics of the cytoplasmic amount of p53 can be neglected. In the nucleus, p53 is transcriptionally active after phosphorylation. Mdm2 must be phosphorylated by Akt [50], and then the phosphorylated Mdm2 may move from cytoplasm to nucleus. Thus, in our model, in the cytoplasm both the phosphorylated and the unphosphorylated Mdm2 are present, while in the nucleus only phosphorylated Mdm2 is considered. Nuclear Mdm2 is responsible for initializing the polyubiquitination of p53 by attaching to it its first ubiquitin moiety [51]. Mdm2 and other enzymes (e.g., p300) catalyze subsequent ubiquitin attachment to p53. Poly-ubiquitinated p53 (by at least four ubiquitin molecules) is then quickly degraded [52], [53]. In our model, at variance of [32], the ubiquitination of p53 is explicitely represented. In a simplified view, we model only the mono- and bi-ubiquitination of p53, and, to take into account the fast degradation of the poly-ubiquitinated species, we assign to the degradation rate of bi-ubiquitinated p53 a value larger than that of the actual rate of the bi-ubiquitinated form, In order to play its role, Mdm2 has to create complexes with p53. We assume that the protection from ubiquitination resulting from the phosphorylation of p53 [54] is mediated by a lower association rate of the complex phosphorylated p53 - Mdm2, and not by a change in the degradation rate. Single- or double-ubiquitinated p53 are deubiquitinated by a protein called HAUSP [55]. In our model, however, we assume that HAUSP level is constant. The model of the nuclear interplay between p53 and Mdm2, described by the equations reported in S1 Text, is thus characterized by a detailed modeling of the mono- and bi-ubiquitination of both p53 and phosphorylated p53, in which the various complexes between Mdm2 and p53 are quantified.

#### Positive feedback loop through PTEN

PTEN hydrolyzes PIP3, produced by PI3K, to PIP2 [31]. PIP3 activates Akt, thus PTEN is an Akt inhibitor. Since Akt phosphorylates Mdm2, a positive feedback loop is enacted where p53 indirectly inhibits its own inhibitor Mdm2. Due to the mediation of the above-mentioned proteins, a significant delay is associated to such a loop. To simplify the model, we assume that the total amounts of PIP (PIP2 + PIP3) and Akt (active+inactive) are constant, and that the binding between PTEN and PIP3, and between PIP3 and Akt, are very rapid. Furthermore, subtle interplays between PTEN and p53 reported in the literature [30] are omitted. Moreover, since the nuclear PTEN is mainly found in well-differentiated and poorly proliferating cells and far more rarely in tumor cells [56], the presence of PTEN in the nucleus is not considered here. The dynamics of this module is ruled by the equations reported in S1 Text.

### Inhibition of Mdm2 by Nutlin

As mentioned above, Nutlin perturbs the p53-Mdm2 system by binding to Mdm2 and occupying the p53 binding pocket on the Mdm2 molecule. As a consequence, Mdm2 cannot form complexes with p53, and p53 ubiquitination is impaired [6]. So, in our model, we assume that each of the Mdm2 forms (unphosphorylated in cytoplasm, and phosphorylated in cytoplasm and nucleus) can be in two states. The first is Mdm2 free from Nutlin, which can bind to p53 and then is called "active". The second one is Mdm2 coupled to Nutlin, so that it cannot form p53-Mdm2 complexes and it is called "inactive". Note that both such states can be phosphorylated or unphosphorylated, and that, when phosphorylated, they can be translocated to or from the nucleus. The accumulation of cytoplasmic inactive Mdm2 is caused by Nutlin binding as well by dephosphorylation of Nutlin-bound phospho-Mdm2. Conversely, its loss derives by Nutlin dissociation, phosphorylation and degradation. These processes yield the equation
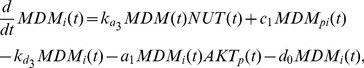
(1)where 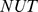 denotes the number of free Nutlin molecules in the cell, 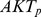 the number of phosphorylated AKT molecules, and the parameters have an obvious meaning. The dynamics of the amount of cytoplasmic inactive phosphorylated Mdm2 is ruled by similar processes, to which nuclear import and export must be added:



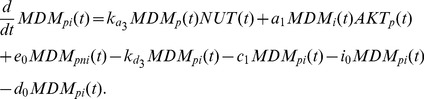
(2)In the nuclear compartment, similarly, Nutlin binds to nuclear-phosphorylated Mdm2 which is inactivated according to the following equations:
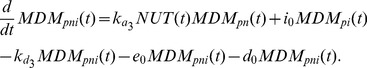
(3)


The dynamic equations for the Nutlin-free cytoplasmic unphosphorylated and phosphorylated Mdm2, as well as for nuclear phosphorylated Mdm2, are reported in S1 Text.

### Nutlin cellular uptake

Although cell uptake of Nutlin appears saturable, the saturation seems to be achieved for rather high extra-cellular concentrations (in HCT116 cells, no clear saturation up to extra-cellular concentrations of 50 microM has been found [57]). In view of the moderate values of the extra-cellular concentration of free Nutlin usually attained in the experiments, we assume a linear uptake. Concerning Nutlin efflux, the export rate is assumed linear for simplicity.

Binding of Nutlin to Mdm2 and the dissociation of their complexes also contribute to the change of the intra-cellular amount of free Nutlin. Taking into account all these processes, for the cell amount of free Nutlin we can write the following equation:
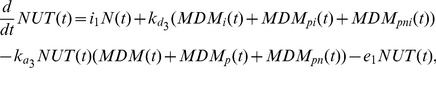
(4)where 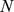 denotes the extra-cellular concentration of free Nutlin.

### Binding to plasma proteins and Nutlin pharmacokinetics in mice

To simulate *in vivo* Nutlin treatments, we exploited the pharmacokinetics data for oral delivery in mice reported in [58] to compute the extra-cellular Nutlin concentration. In particular, we have chosen parameters that fit the measured Nutlin concentration in retina, since the time profile of such a concentration is similar to those in plasma and spleen [58], and then such profile can be taken as an approximation of the pharmacokinetics in the whole organism.

It is important to remark that a substantial binding of Nutlin to plasma proteins has been demonstrated [58], so that the free Nutlin concentration in plasma is only a small fraction of the total Nutlin concentration. In [58], the binding data were fitted to the equilibrium equation
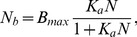
(5)where 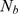 denotes the protein-bound Nutlin concentration, 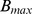 is the concentration of total plasma protein binding sites, and 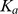 is the equilibrium association constant. From their data, Zhang et al. [58] estimated 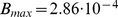
M, and 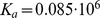
M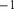. Denoting by 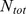 the concentration of total Nutlin, we have
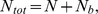
and 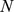 can be expressed in terms of 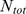, obtaining:



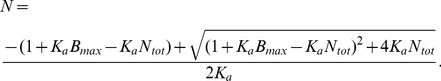
(6)Protein binding is likely to occur also in the retina, and, as suggested in [58], we may assume that in this tissue the binding is the same that in plasma. Therefore, assuming that (i) drug distribution occurs in a single compartment, (ii) only free Nutlin is eliminated, (iii) elimination is linear, and (iv) protein binding is in quasi-steady state, the simplest pharmacokinetic equation for Nutlin reads:
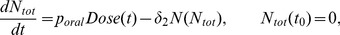
(7)where 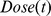 is the drug dose rate (in, e.g., mg Kg^−1^ sec^−1^), 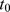 is the initial time of delivery, 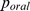 is a factor accounting for the conversion from mg Kg^−1^ to moles, divided by the distribution volume, and 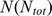 is given by (6). 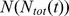 will be the input of Eq. (4) for the intra-cellular Nutlin. Usually, in representing oral delivery, the gastro-enteric release is assumed exponential so that Eq. (7), in case of administration of a single dose at time 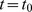, can be rewritten as
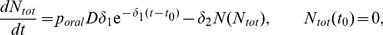
(8)where 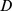 is the dose (in mg Kg^−1^). An easy modification of the above equation accounts for the case of repeated administrations.

## Results

### Comparison with the experimental dose-response curve by Vassilev et al. [6]

**Figure 2:**
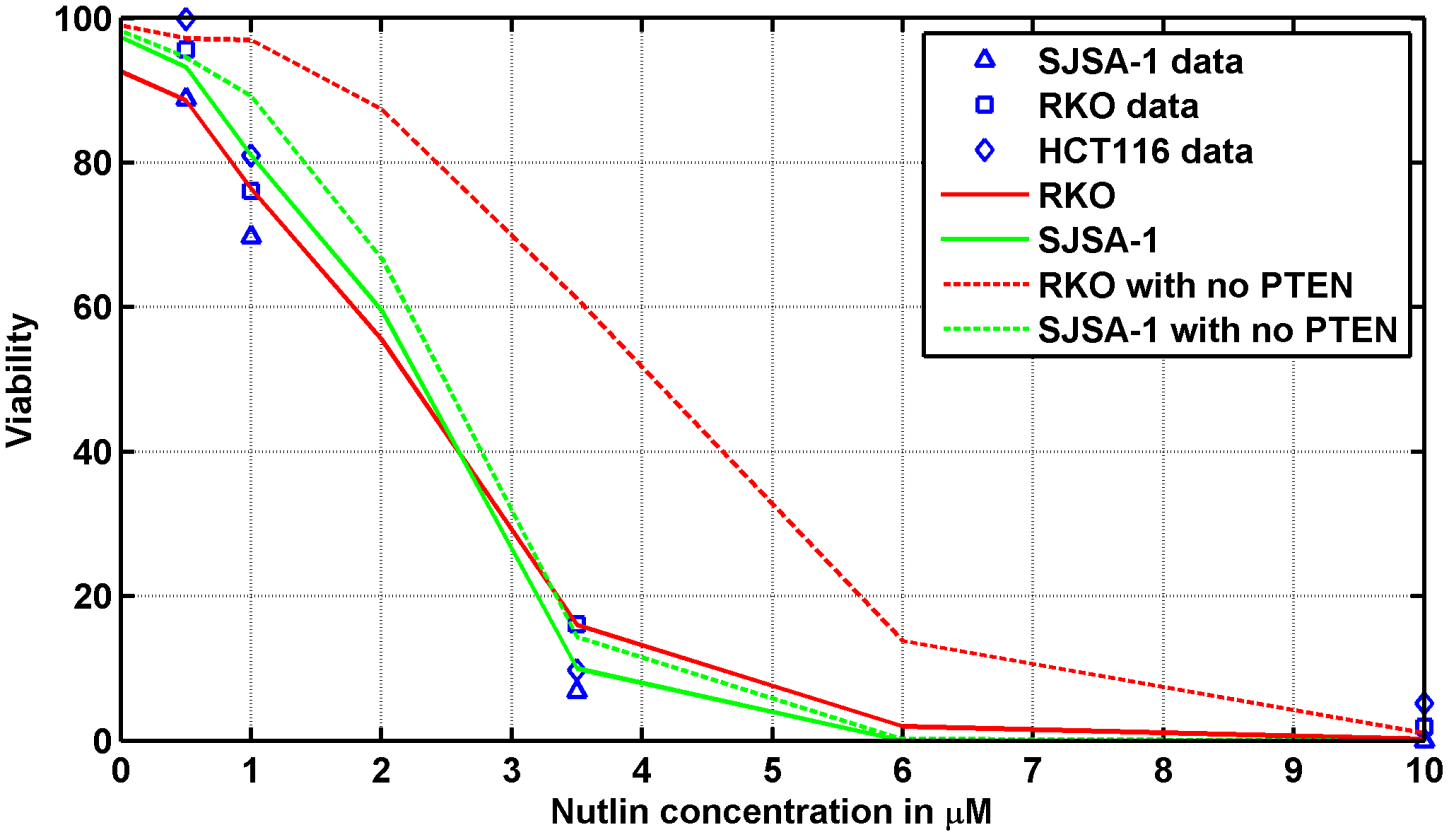
Percentage of cells viable after exposure to different Nutlin concentrations. Symbols: experimental data from Vassilev et al. [6]. Solid and dotted lines: models predictions. In the simulation, cell viability was evaluated 48 h after the start of exposure. The response of 500 cells was simulated for each Nutlin concentration.

In [6], Vassilev et al. reported experimental *in vitro* measurements of cell viability as a function of different concentrations of Nutlin. Different tumor cell lines (HCT116, RKO, SJSA-1) were exposed to Nutlin for five days, and thereafter cell viability was assessed by MTT assay. Since the MTT assay measures the activity of intra-cellular enzymes that reduce the tetrazolium dye, and therefore in a broad sense it measures the cellular metabolic activity, the loss of "viability" according to this assay indicates either cell cycle arrest or cell death. Successive investigations [59], [60] with different experimental techniques demonstrated that Nutlin induces cell arrest in all the considered cell lines, whereas it induces substantial apoptosis (revealed by Annexin V positivity) only in SJSA-1 cells, even though apoptosis is not absent in the other cell lines, particularly in RKO cells. These findings suggested that Nutlin-dependent activation of p53 leads to different outcomes (cell arrest or apoptosis) because of different downstream alterations [59]. The data in [6] show a decrease of the viability when the drug concentration increases, the viability being suppressed at 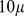
M Nutlin concentration (see Fig. 2). Surprisingly, such a dose-response pattern is quantitatively quite similar for all the considered cell lines despite the fact that the Mdm2 gene is 25-fold amplified in SJSA-1 cells and not amplified in HCT116 and RKO cells [59], [61].

Our stochastic simulations, performed with the parameter values reported and discussed in S3 Text (see Tables 1 and 2 in S3 Text) show that the proposed model is nicely able to reproduce these experimental curves, as illustrated in Fig. 2, by assuming that the loss of "viability" is caused by the rising of p53 level over a certain threshold [62]. More precisely, we assume that if the amount 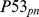 of nuclear-phosphorylated p53 exceeds a *threshold* value 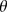 for at least a time 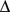, then cell arrest or apoptosis is triggered, and the viability of that cell is lost.

The adequacy of this strict assumption in fitting the data in [6] might be questioned when the response of a substantial fraction of cells consists only in cell cycle arrest (as for HCT116 and RKO cells), since there are several evidences that the Nutlin-induced cell cycle block is reversible after the end of the stimulus, and that the kinetics of this recovery is different in different cell lines [60], [63], [64], [65]. Concerning RKO cells, it has been suggested that in cells not undergoing apoptosis the cell cycle block may be quite long [60]. On the contrary, contrasting results on the action of Nutlin on HCT116 cells have been found [60], [64], [65], with evidences that the time needed to recover proliferation after treatment may be very short (full proliferation was observed three days after the removal of Nutlin [60]) or rather long (colony formation was totally suppressed by Nutlin treatment for seven days after drug removal [65]). Therefore, taking into account the lack of consensus on the recovery kinetics of arrested HCT116 cells, we restricted ourselves to fit only the data from RKO and SJSA-1 cells.

To obtain Fig. 2, we tried 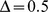 and 1 h with few changes in the numerical results, and selected 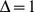 h for the final fitting. To predict the fraction of viable cells at each concentration, the individual response of 500 cells was simulated (see S2 Text for details on the simulation algorithm). The number of Mdm2 gene copies 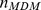 was assumed equal to 2 when data from RKO cells were fitted, and equal to 50 in the case of data from SJSA-1 cells. Different values of the p53 threshold were allowed for Mdm2-amplified and non-amplified cells. The values of the parameters of Table 2 of S3 Text, together with the threshold values, were adjusted by a trial-and-error procedure. As expected, in the case of SJSA-1 cells, a threshold value much lower than the value set for RKO cells (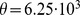 vs. 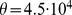 molecules/cell) was needed, to compensate for the lower p53 levels imposed by the abundance of Mdm2 molecules. Actually, other causes such as differences in the p53 transcriptional activity or in the abundance of downstream molecules can contribute to set the threshold value, but for sake of simplicity and since there are no clear experimental evidences, they are not included in the present model.

Note that the dose-response data reported in [6] are given as a function of the total Nutlin concentration in the medium. Some degree of Nutlin binding to the culture medium proteins, however, has been demonstrated in [58], and from such measurements we could estimate the equilibrium association constant and the concentration of medium binding sites (see Fig. 1 and Table 3 of S3 Text). Supposing that the binding capability of the medium employed in [6] and of the medium employed in [58] be the same, we computed for each total concentration the corresponding concentration of free Nutlin by means of (6). These values were used in Eq. (4) to calculate the intra-cellular free Nutlin amount.

**Figure 3:**
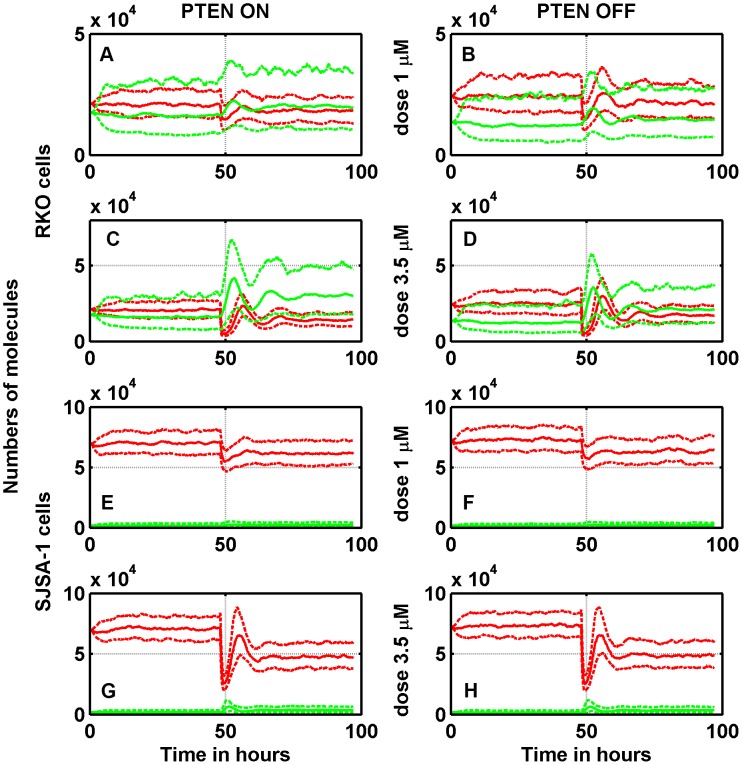
Time-courses of the phosphorylated nuclear p53 (green) and nuclear Mdm2 (red) in response to two different Nutlin extra-cellular concentrations in RKO and SJSA-1 cells. Solid lines represent medians, and dotted lines first and third quartiles. The Nutlin exposure begins at 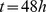. Left column: PTEN feedback loop is active; right column: PTEN feedback loop is inactive.

**Figure 4:**
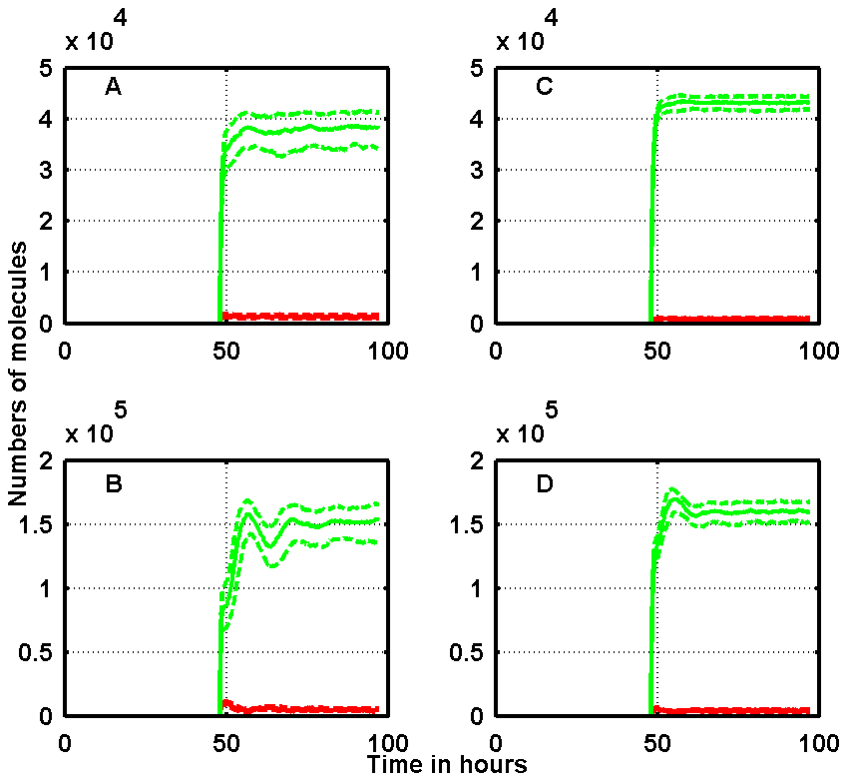
Time-courses of the total (green) and free (red) intracellular Nutlin in response to two different Nutlin extra-cellular concentrations in RKO (left column) and SJSA-1 (right column) cells. Extra-cellular concentrations: panels A and C, 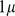
M; panels B and D, 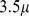
M. Solid lines represent medians, and dotted lines first and third quartiles. The PTEN feedback loop is active.

We also predicted the cell response when the PTEN feedback was disabled (keeping all the other parameters unchanged), to mimic tumor cells in which PTEN is not expressed. The expected reduction of Nutlin efficacy occurs only when no amplification of Mdm2 is present, when 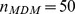, instead, the effect of PTEN deletion is very limited. It is interesting to compare the dynamics of nuclear-phosphorylated p53 and nuclear-phosphorylated Mdm2 after the exposure to different Nutlin concentrations among those of Fig 2. In Fig. 3, we show stochastic simulations of RKO cells for the exposure to total concentrations of 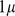
M and 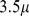
M (panels A–D). The median number of p53 molecules grows after the start of Nutlin exposure and tends to stabilize after some oscillations to a value higher than the baseline value. When the PTEN feedback is disabled, the increase of p53 amount is reduced. In the panels E–H of Fig. 3, showing the simulation of SJSA-1 cells, we may note that the number of nuclear Mdm2 molecules is larger than in the case of RKO cells, and the number of p53 molecules is smaller, in agreement with the presence of a robust Mdm2 gene amplification. Fig. 4 shows the corresponding dynamics of total and free intra-cellular Nutlin. We recall here that 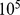 molecules in a cell of volume equal to 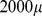
m^3^ correspond to a concentration of 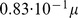
M. Note that most of the intra-cellular Nutlin is bound to the Mdm2 molecules.

### In silico dose-response curve after Nutlin delivery in mice

**Figure 5:**
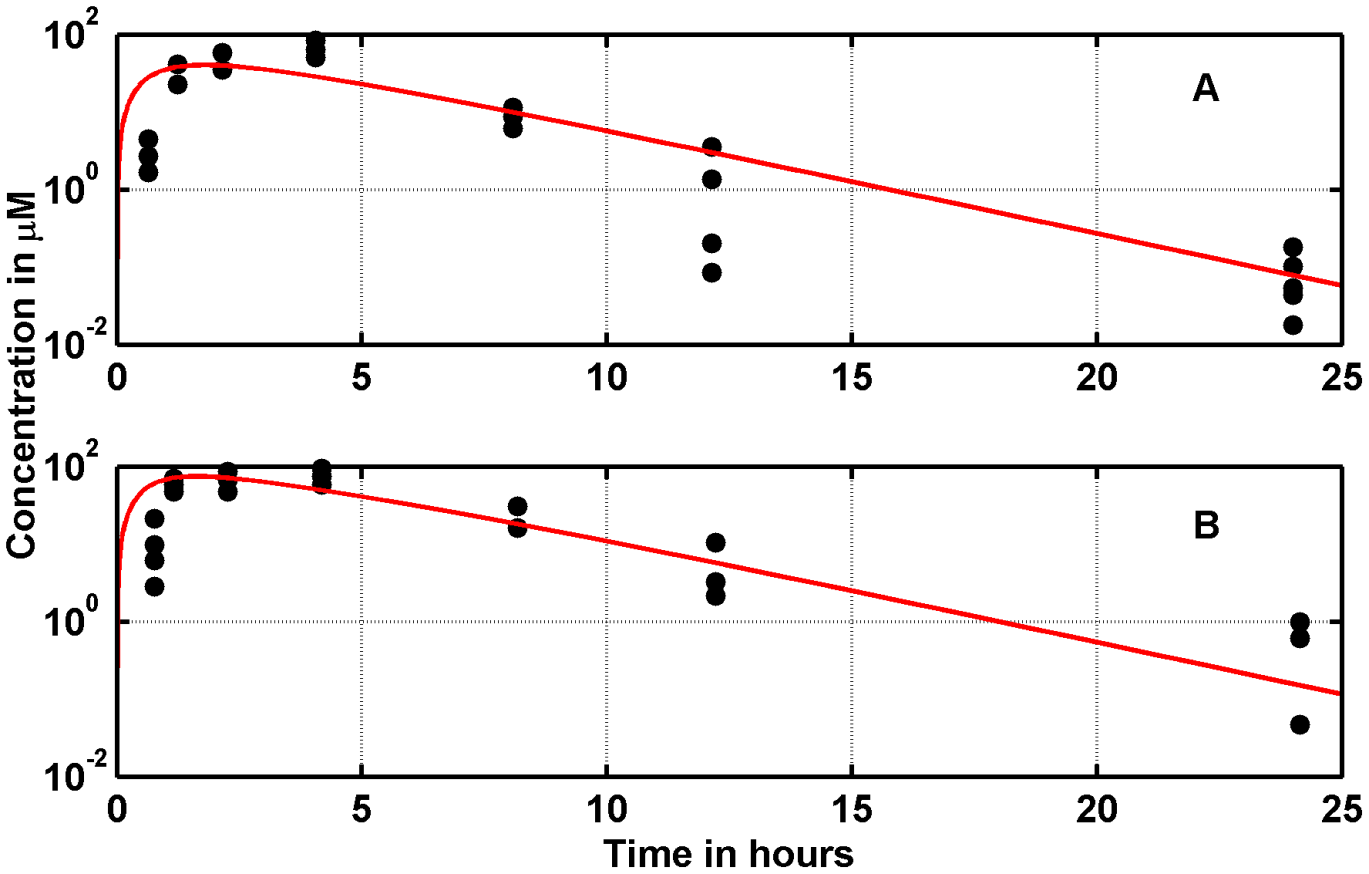
Nutlin pharmacokinetics: Data from Zhang et al. [58] (total Nutlin concentration in mouse retina after oral delivery, black dots), and fittings by the model (8). Doses: panel A, 100 mg/Kg; panel B, 200 mg/Kg.

In Vassilev's experiments [6], the extra-cellular Nutlin concentration was maintained constant. This is not the case of *in vivo* delivery, when the drug is given as boli, i.e., computationally speaking, in impulsive doses. By fitting available data of Nutlin pharmacokinetics [58], we identified the parameters of the pharmacokinetic model (8) (see Fig. 5, panels A and B, and Table 3 of S3 Text). By means of that model, we have simulated realistic oral deliveries of Nutlin in mice.

Since during bolus delivery cells are transiently exposed to the drug, the possible cell recovery from cycle arrest is expected to have a remarkable influence on the fraction of cells still blocked at the assessment time. Some exploratory simulations (see S4 Text), where recovery is allowed to occur a random time since active p53 level drops below the threshold, confirm the extent of this impact, showing the great importance of the mean recovery time and of the time at which "viability" is assessed. Thus, on the basis of our simple hypothesis on the cell response to Nutlin, we can predict the fraction of cells that *do not respond* during the time of simulation, whereas we cannot predict in principle the fraction of cells that are blocked (or are in apoptosis) at the end of simulation. Of course, these quantities coincide if cells preferentially undergo apoptosis (SJSA-1 cells), or if the recovery time is larger than the interval between the start of treatment and the assessment time, as it should be for RKO cells.

**Figure 6:**
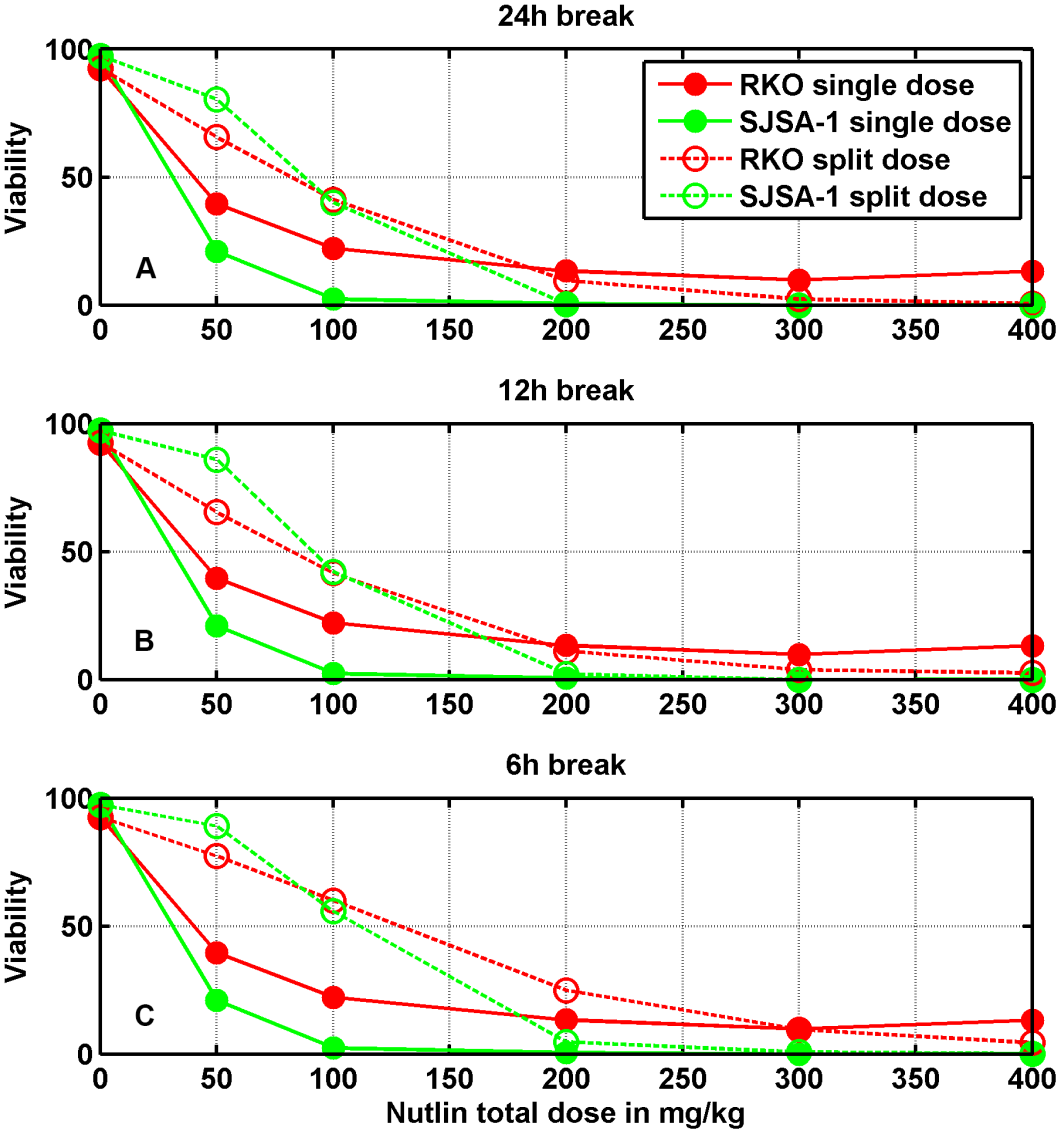
Prediction of the percentage of cell viability after oral dosage in mice. Filled symbols correspond to single dose delivery (in all panels), open symbols to the case in which the total dose was split into four equal doses given with 24 h breaks (panel A), 12 h breaks (panel B), and 6 h breaks (panel C). Cell viability was assessed 120 h after the first dose.

In Fig. 6, we show the simulated dose-response curves in the case of a dose given as a single bolus (solid lines), and when the dose is split in four boli (dashed curves), administered with 24 h (panel A), 12 h (panel B), and 6 h breaks (panel C).

The dose-response curves are deeply affected by the splitting. Indeed: i) both in RKO cells and in SJSA-1 cells, splitting the dose causes a larger viability up to about 200 mg/kg; ii) in SJSA-1 cells, doses larger than 200 mg/kg guarantee almost zero viability for both the single and the split dose delivery; iii) in RKO cells, for doses larger than 200 mg/kg split doses are more effective than the single dose, which keeps a residual fraction of non-responding cells of about 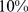 at 400 mg/kg; iv) when the fractionated doses are delivered with intervals of 6 h, the viability is generally larger than in the case of 24 h intervals, i.e., the therapeutic response is disadvantaged; v) after a single dose, SJSA-1 cells appear more responsive than RKO cells.

**Figure 7:**
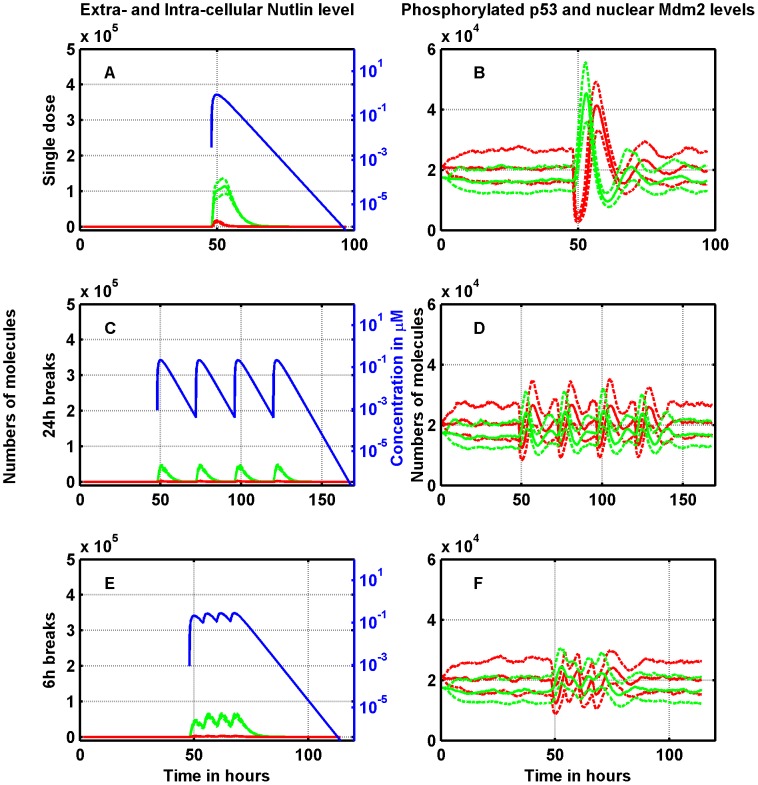
Single-dose vs. split delivery of a total dose of 50 mg/kg of Nutlin: Effects on the RKO cell line. Left panels: dynamics of Nutlin. Extra-cellular free concentration (blue, on the log scale), total intra-cellular amount (green), free intra-cellular amount (red). Right panels: time-course of p53 (green) and Mdm2 (red). Upper panels: single dose. Central panels: the dose is split in four doses delivered with an interval of 24 hours in between. Lower panels: again the dose is split in four doses, but here the time between two doses is 6 hours. Solid lines represent medians, and dotted lines first and third quartiles.

**Figure 8:**
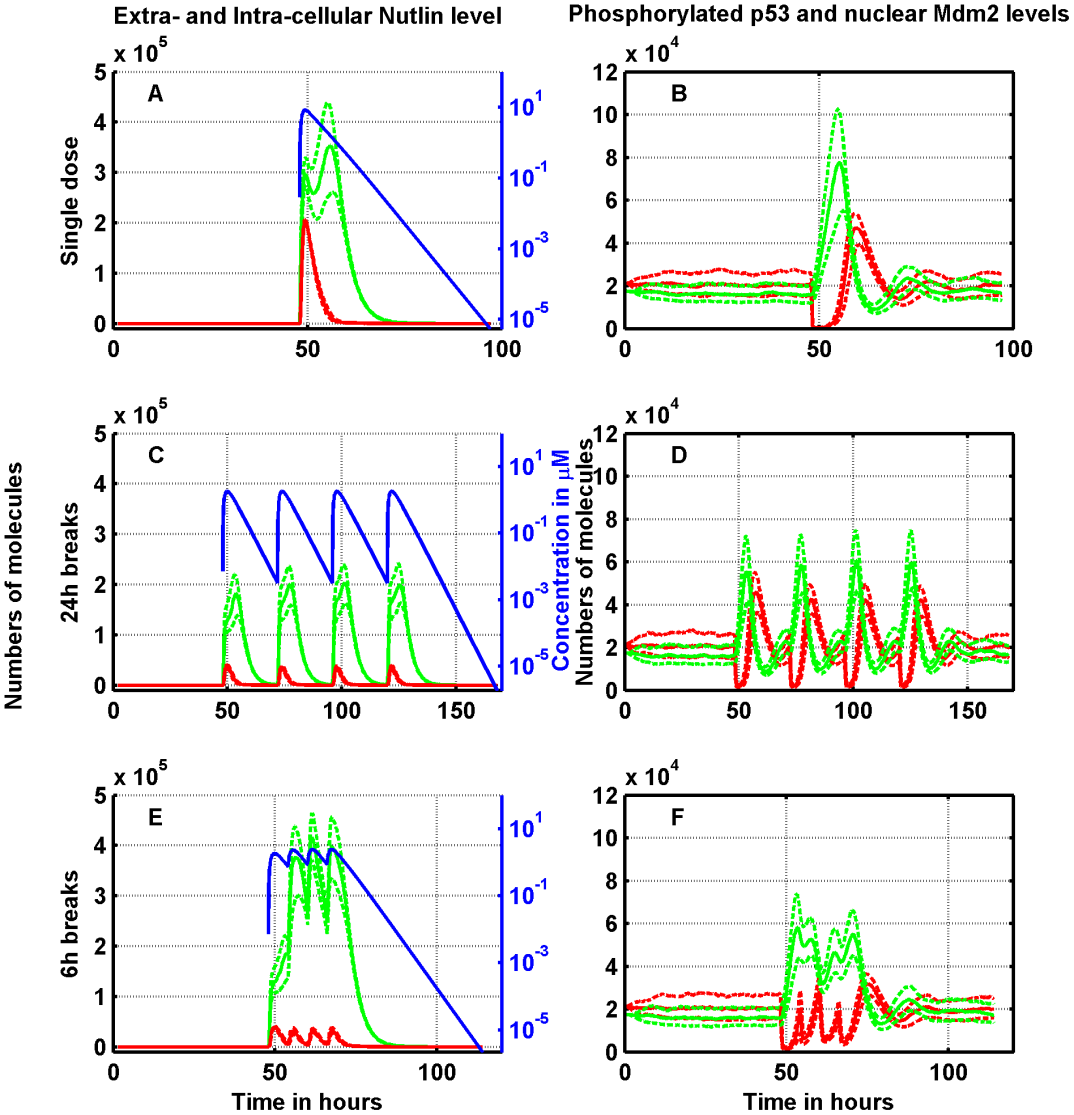
Single-dose vs. split delivery of a total dose of 400 mg/kg of Nutlin: Effects on the RKO cell line. Left panels: dynamics of Nutlin. Extra-cellular free concentration (blue, on the log scale), total intra-cellular amount (green), free intra-cellular amount (red). Right panels: time-course of p53 (green) and Mdm2 (red). Upper panels: single dose. Central panels: the dose is split in four doses delivered with an interval of 24 hours in between. Lower panels: again the dose is split in four doses, but here the time between two doses is 6 hours. Solid lines represent medians, and dotted lines first and third quartiles.

Some insights into the above behavior can be obtained by analyzing the dynamics of extra-cellular free Nutlin and intra-cellular Nutlin, both total and free, and the time-courses of nuclear p53/Mdm2. Figs. 7 and 8 report such profiles in RKO cells when the total Nutlin dose is 50 mg/Kg and 400 mg/Kg, respectively. In Figs. 7B and 8B, the response to the single doses is shown by plotting the profiles of nuclear-phosphorylated p53 and of nuclear-phosphorylated Mdm2. Note that although immediately after the dose delivery the Mdm2 amount reduces close to zero, after a short time-lag the number of molecules rapidly recovers and a high peak is reached 10 hours after the drug administration. Concerning p53, the peak is reached before Mdm2 regrows over the baseline value, and ultimately also p53 is restored to its pre-delivery value. Note, moreover, that the p53/Mdm2 response is initiated by the first small peak of the total intra-cellular Nutlin amount (well visible in Fig. 8A) corresponding to the peak of extra-cellular free Nutlin concentration, and not by the delayed and dominant peak of intra-cellular Nutlin. Due to the rapid drug elimination, the splitting with 24 h breaks results in four almost independent dynamics. In such a case, there is only a slight accumulation of the total intra-cellular Nutlin, more visible at 400 mg/kg (see Fig. 8C), which is mirrored in the nuclear p53 peaks (see Fig. 8D). When the interval among split doses is 6 h, instead, there is a clear accumulation of the total intra-cellular Nutlin both at doses of 50 and 400 mg/Kg. Quite surprisingly, the p53 peaks, although rather merged together, have heights on average smaller than the peaks achieved by 24 h breaks (compare Fig. 7D and F, and Fig. 8D and F).

The figures also evidence a nonlinear relationship between the dose injected and the total amount of intra-cellular Nutlin, and between the dose injected and the peak of nuclear p53 (measured from the baseline value). When the dose of 400 mg/Kg is split with 24 h breaks, the first median p53 peak is over 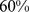 of the median p53 peak after a single dose, whereas the dose injected is only 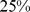 (compare Fig. 8B and D). The nonlinearity is present also at the dose of 50 mg/kg but it is less pronounced: after splitting with 24 h breaks, the median p53 peaks are about 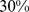 of the peak after the single dose (compare Fig. 7B and D). The complex p53 regulation network, may be the sources of this nonlinearity.

The inefficacy of dose-splitting at low doses and the opposite behavior at high doses (found in simulating RKO cells) can be explained by the contrasting effects of the following factors: a) the existence of a threshold triggering the cell-arrest/apoptotic response penalizes the dose fractionation, unless the dose is very high. In fact, if the p53 level exceeds the threshold after a single dose delivery, the same is not granted for split doses; b) the nonlinearity between dose and intra-cellular Nutlin amount (above described) rewards fractionated schedules by advantaging small doses. The necessity that the level of 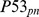 crosses a threshold to elicit the cell response also explains the general increase of viability observed in our simulations with 6 h breaks, since in this case the p53 peaks were smaller than the p53 peaks found with 24 h-break splitting. A finer inspection of the simulated response shows that in the case of splitting with 6 h breaks, the PTEN amount reaches the largest value (not shown), likely because the level of active p53 is rather sustained over the whole 24 h period of Nutlin administration (we remind that, according to the model, the overall transcription activity of p53 is substantially related to the time integral of its concentration). The high level of PTEN causes a reduced Mdm2 phosphorylation and then accumulation of Nutlin-bound non-phosphorylated Mdm2 in the cytoplasm. In this way the accumulation of total intra-cellular Nutlin during drug delivery with 6 h breaks may not translate into a corresponding increase of the peak level of nuclear active p53.

Although the response of RKO and SJSA-1 cells to a continuous Nutlin exposure is quite similar (see Fig. 2), SJSA-1 cells are predicted to be more responsive than RKO cells after a single bolus delivery (Fig. 6). We may advance an explanation based on the role of PTEN. First, note that the relationship between the dose-response curves of the different cell lines with PTEN OFF in Fig. 2 is similar to that of Fig. 6, single dose, i.e., SJSA-1 cells result more responsive. With PTEN ON and continuous exposure to the drug, the slow positive PTEN feedback loop has time to play its role and, as a result, the active p53 level increases in the nucleus. This favors the RKO "mortality" but not that of SJSA-1 cells. Indeed, the strong Mdm2 overexpression makes the positive feedback promoted by PTEN less important. Thus, we can see a viability difference between PTEN ON and OFF for RKO, but not for SJSA-1 cells. In the case of a dose delivered with a single bolus, the input signal does not last long enough to trigger the positive PTEN feedback, so RKO cells do not exhibit Mdm2 blocking in cytoplasm and the consequent increase of nuclear p53. Their viability thus remains greater than that of SJSA-1 cells.

## Discussion

Although deterministic models give valuable information on the average behavior of a biochemical system, they are by definition unable to reproduce statistical behavior differences, both intra-cellular (i.e., possible random changes in the response of single cells when observed for a long time) and inter-cellular (i.e., different responses of two "identical" cells). The experimentally observed dose-response curves mirror this inter-cellular variability of the response to drug delivery. In recent years a vast body of research has focused on the randomness affecting biomolecular networks. Two kinds of stochasticity are usually considered. The first kind is caused by the interplay between cells and their microenvironment. This stochasticity is termed extrinsic noise. Another kind of randomness comes from the intrinsic stochastic nature of chemical reactions, and its effect becomes more evident when the number of transcripts or proteins is low. In such a case, differential equations do not allow an accurate representation of the dynamics of those transcripts and/or proteins. Interestingly, even when a differential equation model appears to be feasible, in some conditions the average behavior of its stochastic counterpart can diverge significantly from the deterministic prediction (see e.g., [66]). However, another internal source of noise is often neglected: the randomness of the process of gene activation/deactivation. Actually this kind of noise might be one of the major sources of random fluctuations in intra-cellular protein concentrations. If the switching rates of the genes are very large, one can neglect this noise because it is 'filtered' by the network itself. Still, this is not possible in many cases as it has been experimentally shown [37], [38], [39] and theoretically confirmed [40], [41], [42], [43].

In reproducing the original Vassilev's experiments [6] *in-silico*, our numerical simulations have shown that the stochasticity in p53, Mdm2 and PTEN regulation, introduced at the level of gene transcription, leads to a large variability of the cell response to Nutlin, with the fraction of responding cells growing with the Nutlin dose. As far as the feedbacks are concerned – feedback is the other key concept of Systems Biology – our simulations suggest that the positive feedback enacted by means of PTEN-Akt-PIP might be essential to reproduce the dose-response of RKO cells, whereas it might only have a negligible effect in SJSA-1 cells. This would imply that the ability of the loop in sequestering Mdm2 in the cytoplasm is no longer sufficient to significantly impact on the Mdm2-p53 dynamics when Mdm2 is strongly amplified.

Our condition about the loss of viability, based on [62], depends on the phosphorylated p53 level in nucleus, which implies we consider only transcription-dependent mechanism of p53-dependent apoptosis. Indeed, p53 can trigger apoptosis also in a transcription-independent manner. Mono-ubiquitinated p53 can be transported to the mitochondria where it interacts with BCL2 family proteins, so to activate Bak [67] or release Bax from the complexes with BCL-2/XL [68]. This in turn leads to the mitochondrial membrane permeabilization, cytochrome-c release, and caspase cascade activation. However, in Nutlin-based mono-therapy, where p53 activation is achieved by a mechanism different from that triggered by DNA damage, we can expect that the dynamics of mono-ubiquitinated p53 closely follows the dynamics of phosphorylated nuclear p53. So, transcription-independent apoptosis should likely parallel transcription-dependent apoptosis. In case of combined therapy, instead, the uniqueness of such a dynamics is expected to be broken, and our model should be completed with the mitochondrial function of p53, following e.g. [67].

Concerning the clinical applicability of Nutlin, our simulations of boli-based drug delivery suggest that remarkable effects on cell viability may be observed only when large doses are administered, and that dose-splitting generally worsens the response at low-medium doses. Of course, these indications are only preliminary, and simulations of a wider set of patterns of fractionated delivery would be valuable. More importantly, the study should be completed by the evaluation of the toxicity of different schedules. The reported results, in addition to the relatively short clearance time of Nutlin, might cast some doubts on the use of Nutlin in clinics as a monotherapy for cancers associated to the cell lines here considered. However, the recently proposed role of Nutlin as a "neutralizer" of chemoresistance to other antitumor drugs [13], [14], [15] might open a new way for the clinical use of this agent.

Furthermore, we also wish to stress that viability predictions when cells are mainly affected by cell cycle arrest should require accurate information on the kinetics of the possible return into the cell cycle. In such a case, the model predictions on the efficacy of fractionated schedules could actually be experimentally validated, provided that careful experimental assessment of the pharmacokinetics and of the binding properties of the drug be available.

Small agents different from Nutlin that reversibly inhibit the capability of Mdm2 to bind p53 have been recently discovered. Examples of such agents are MI-219 [69], RG7112 [70] and RG7388 [71]. After suitable parameter tuning, our model could be applied to describe the action of all these new anticancer chemicals.

Although quite detailed, the present investigation has some limitations and misses a number of possibly important points. We wish to discuss some of them in the following.

First, the role of MDMX in p53 regulation has not been explicitly described in our model. MDMX protein is an antagonist of p53 with some similarity to Mdm2 [72]. When overexpressed, MDMX impairs the activity of Nutlin in increasing the p53 level [73]. MDMX binds p53, with the consequent possible inactivation of that protein, and the p53 binding is not inhibited by Nutlin [73]. Although MDMX does not directly exert E3 ligase activity, it forms heterodimers with Mdm2 enhancing the efficiency of Mdm2 in p53 polyubiquitination [74]. However, p53 is not a transcription factor for the MDMX gene [72], so that, strictly speaking, there is no negative feedback in the p53 regulation through MDMX. As a consequence, the impact of the relative abundancy of MDMX in different cell lines may be (and implicitly is) assessed in our model by adjusting the values of some parameters, such as the p53 ubiquitination rate. It is worth mentioning that in both HCT116 and SJSA-1 cell lines, MDMX is poorly expressed [75], [76]. Anyway, including MDMX among the players of the model would increase its flexibility, also allowing for the quantification of the action of MDMX-targeting drugs [77], [78].

An additional negative feedback involving p53 can be mediated by Wip1. Wip1 is transcriptionally dependent on p53 and other factors like CREB and NF-kB, and its role is to shut down the p53 regulatory unit after the repair of double strand breaks (DSBs) is successfully completed [79], [80]. Basically, it dephosphorylates p53 and returns Mdm2 to the active form after its deactivation by ATM which is induced by DSB occurrence. Since Wip1 is mainly produced after DSB formation while it remains at low level when DNA is intact [79], it should not significantly influence the pharmacodynamics of Nutlin in the case of monotherapy. This guess has been experimentally confirmed in the U2-OS cell line, comparing the response to Nutlin of Wip1-silenced and normal cells (K. Szoltysek and P. Janus, personal communication). Thus Wip1 was not included in the model. The extension of the model to include the Wip1 feedback would be on the contrary very important to describe the effects of the combination of Nutlin treatment and radiotherapy.

In recent years, an increasing number of miRNAs [81] have been shown to be involved in shaping p53 functions and p53 regulation, and a very complex network of interactions is likely to be unveiled [82], [83]. A large part of such miRNAs, e.g., the miR-34 family and the miR-15/16 family, are involved in the downstream functions of p53, such as cell arrest and apoptosis, but miRNAs also appear to exert some control on the p53 expression. For instance, it has been observed that miR-125b [84] and miR-504 [85], when overexpressed, keep the p53 level low by direct binding to the p53 mRNA. In our model, under the assumption of a constant level of the above-mentioned miRNAs during Nutlin treatment, their action should just result in the modulation of the values of parameters such as the p53 translation rate and the p53 mRNA degradation rate. Other miRNAs may act more subtly by establishing positive feedbacks, e.g., the loop connecting miR-34a, SIRT1 and p53, or miR-192/194, Mdm2 and p53 [83]. By impairing the expression of Mdm2, overexpression of miR-192/194 has been reported to potentiate the efficacy of the Mdm2 inhibitor MI219 [86]. Although these findings are of great interest, we believe that further studies are needed to elucidate the relative importance of these controls for p53 biology before including miRNA-mediated feedbacks in our model of p53 regulation. Moreover, we remark that up to now only a limited number of investigations is available to assess the values of the biochemical parameters characterizing the activity of miRNAs. We also mention that the same miRNA may exert opposite functions in different tumors: for instance, the overexpression of miR-125b that down-regulates p53 in colorectal cancers [87], seems to induce cell cycle arrest and apoptosis in Ewing sarcoma cells, possibly by p53 activation through down-regulation of PI3K and phospho-AKT [88].

Turning now to model features more directly linked with Nutlin activity, the cellular influx and efflux of Nutlin might be modeled in more detail. For example, there is preliminary evidence that Nutlin can be a substrate for ABC transporters like p-glycoprotein [89], which suggests the possibility of a nonlinear efflux rate. Further quantitative experimental information, however, is required to implement this effect in our model.

We finally remark that a biomolecular network is never isolated, and its dynamics may be deeply affected by its interactions with many other (often unknown) networks, as well as by various random signals coming from the extra-cellular environment. These "extrinsic noises" may substantially synergize with the intrinsic and the gene-regulation stochasticity [90]. For example, since it is not clear the mechanism setting the p53 threshold triggering the cell response – such a mechanism can actually depend on the transcriptional activity of p53 and on the interplay of a number of other genetic networks – it would be of interest to model this threshold as an average value perturbed by bounded extrinsic noise. In addition, the pharmacokinetics of many drugs can be perturbed by random fluctuations of the clearance rate, affecting sometimes in turn the pharmacodynamics, as investigated in [91] for generic non-targeted chemotherapies in macroscopic solid tumors. Moreover, the dynamics of biomolecular networks can be influenced by the spatial diffusion of chemicals in the intra-cellular space. Diffusion may lead to peculiar phenomena of significant biological impact [92], [26], revealing important synergies with the temporal stochasticity [93]. These issues are worth investigating in connection with the cell response to Nutlin, and we intend to face them in the near future.

## Supporting Information

S1 Text Dynamics of p53 regulation: equations of p53-Mdm2 module and equations of PTEN-PIP3-Akt module.
(PDF)

S2 Text Hybrid stochastic algorithm and its implementation.
(PDF)

S3 Text Parameters and their justifications. The rationale of parameter choice is provided. Parameter values are given in Tables 1, 2, and 3. Table 1 reports values taken from the literature, Table 2 values heuristically chosen to fit *in vitro* dose-response data from Vassilev *et al.* [6], Table 3 values of parameters characterizing Nutlin pharmacokinetics in mice.
(PDF)

S4 Text Effect of recovery of proliferation after Nutlin-induced cell cycle arrest.
(PDF)
