# Correction: The Pharmacodynamics of the p53-Mdm2 Targeting Drug Nutlin: The Role of Gene-Switching Noise

**DOI:** 10.1371/journal.pcbi.1004215

**Published:** 2015-03-17

**Authors:** The PLOS Computational Biology Staff

There is an error in the funding statement of this paper. KP received a grant from the Polish National Center for Science (decision number: DEC-2012/05/D/ST7/02072), and the sentence ‘No funds were given to KP for publication fees’ should not be included.
