# Supplementary material for: The Pharmacodynamics of the p53-Mdm2 Targeting Drug Nutlin: The Role of Gene-Switching Noise
Source: PLoS Comput Biol. 2014 Dec 11;10(12):e1003991. doi: 10.1371/journal.pcbi.1003991 (PMC4263360; doi:10.1371/journal.pcbi.1003991)
Supplement: S1 Text — Dynamics of p53 regulation: equations of p53-Mdm2 module and equations of PTEN-PIP3-Akt module. (PDF) [file pcbi.1003991.s001.pdf]

Supporting Information file S1

October 28, 2014

S1. Dynamics of p53 regulation

In this supplementary material file, we write the ordinary differential equations that, together with those described in the main text, rule the deterministic dynamics of all involved macromolecular species. We recall that the deterministic part of our model is coupled to the stochastic processes $G_{P53}(t)$, $G_{MDM}(t)$, and $G_{PTEN}(t)$ (see Section 2.1 of the main text), and *vice-versa*. Below we summarize the variables of the mathematical model. Apart from N, which is a concentration, the other symbols denote the intracellular number of molecules of the corresponding chemical species.

- N - Nutlin extracellular concentration
- NUT - intracellular Nutlin
- $P53_{RNA}$ - p53 transcript
- MDM_{RNA} - Mdm2 transcript
- $PTEN_{RNA}$ - PTEN transcript
- MDM - cytoplasmic Mdm2
- MDM_p - phosphorylated cytoplasmic Mdm2
- MDM_{pn} - phosphorylated nuclear Mdm2
- MDM_i - cytoplasmic Mdm2 inactivated by Nutlin binding
- MDM_{pi} - phosphorylated cytoplasmic Mdm2 inactivated by Nutlin binding
- MDM_{pni} - phosphorylated nuclear Mdm2 inactivated by Nutlin binding
- $P53_n$ - unphosphorylated nuclear p53 dimers
- $P53_{pn}$ - phosphorylated nuclear p53 dimers
- $P53_n|MDM_{pn}$ - complex between MDM2pn and unphosphorylated nuclear p53 dimers
- $P53_{pn}|MDM_{pn}$ - complex between MDM2pn and phosphorylated nuclear p53 dimers
- $P53_n^u$ - single-ubiquitinated unphosphorylated nuclear p53 dimers
- $P53_n^{uu}$ - double-ubiquitinated unphosphorylated nuclear p53 dimers
- $P53_{pn}^u$ - single-ubiquitinated phosphorylated nuclear p53 dimers
- $P53_{pn}^{uu}$ - double-ubiquitinated phosphorylated nuclear p53 dimers

- $P53^u_n|MDM_{pn}$ - complex between MDM2pn and single-ubiquitinated unphosphorylated nuclear p53 dimers
- $P53^u_{pn}|MDM_{pn}$ - complex between MDM2pn and single-ubiquitinated phosphorylated nuclear p53 dimers
- $PTEN$ - cytoplasmic PTEN
- PIP_p - phosphorylated PIP3
- PIP_{tot} - unphosphorylated + phosphorylated PIP3
- AKT_p - phosphorylated Akt
- AKT_{tot} - unphosphorylated + phosphorylated Akt

Equations of p53-Mdm2 module

p53 transcript. Transcription − degradation:

$$\frac{d}{dt}P53_{RNA}(t) = s_2 G_{P53}(t) - d_8 P53_{RNA}(t). \tag{1}$$

Mdm2 transcript. Transcription − degradation:

$$\frac{d}{dt}MDM_{RNA}(t) = s_0 G_{MDM}(t) - d_6 MDM_{RNA}(t). \tag{2}$$

Nuclear p53. Translation + dephosphorylation − phosphorylation + Mdm2-p53 complex dissociation − Mdm2-p53 complex formation − degradation + deubiquitination of mono-ubiquitinated p53:

$$\begin{aligned}\frac{d}{dt}P53_n(t) = {}& t_2 P53_{RNA}(t) + c_0 P53_{pn}(t) - a_0 P53_n(t) + k_{d_1} P53_n|MDM_{pn}(t) \\ & - k_{a_1} MDM_{pn}(t) P53_n(t) - d_2 P53_n(t) + k_{d_u} P53^u_n(t).\end{aligned} \tag{3}$$

Nuclear phospho-p53. Phosphorylation − dephosphorylation − degradation − Mdm2-p53 complex formation + Mdm2-p53 complex dissociation + deubiquitination of mono-ubiquitinated phospho-p53:

$$\begin{aligned}\frac{d}{dt}P53_{pn}(t) = {}& a_0 P53_n(t) - c_0 P53_{pn}(t) - d_3 P53_{pn}(t) - k_{a_2} MDM_{pn}(t) P53_{pn}(t) \\ & + k_{d_1} P53_{pn}|MDM2_{pn}(t) + k_{d_u} P53^u_{pn}(t).\end{aligned} \tag{4}$$

Complex between p53 and nuclear phospho-Mdm2. Complex formation − complex dissociation (with or without ubiquitination):

$$\frac{d}{dt}P53_n|MDM_{pn}(t) = k_{a_1} MDM_{pn}(t) P53_n(t) - (k_u + k_{d_1}) P53_n|MDM_{pn}(t). \tag{5}$$

Complex between phospho-p53 and nuclear phospho-Mdm2. Complex formation − complex dissociation (with or without ubiquitination):

$$\frac{d}{dt}P53_{pn}|MDM_{pn}(t) = k_{a_2} MDM_{pn}(t) P53_{pn}(t) - (k_u + k_{d_1}) P53_{pn}|MDM_{pn}(t). \tag{6}$$

Complex between mono-ubiquitinated p53 and nuclear phospho-Mdm2. Complex formation − complex dissciation (with or without ubiquitination):

$$\frac{d}{dt}P53^u_n|MDM_{pn}(t) = k_{a_1} MDM_{pn}(t) P53^u_n(t) - (k_u + k_{d_1}) P53^u_n|MDM_{pn}(t). \tag{7}$$

Complex between mono-ubiquitinated phospho-p53 and nuclear phospho-Mdm2. Complex formation – complex dissociation (with or without ubiquitination):

$$\frac{d}{dt}P53^u_{pn}|MDM_{pn}(t) = k_{a_2}MDM_{pn}(t)P53^u_{pn}(t) - (k_u + k_{d_1})P53^u_{pn}|MDM_{pn}(t). \quad (8)$$

Mono-ubiquitinated p53. Mdm2-p53 complex dissociation with ubiquitination + mono-ubiquitinated p53 with nuclear phospho-Mdm2 complex dissociation without ubiquitination + deubiquitination of double-ubiquitinated p53 + dephosphorylation – phosphorylation – mono-ubiquitinated p53 with nuclear phospho-Mdm2 complex formation – degradation – deubiquitination:

$$\begin{aligned}\frac{d}{dt}P53^u_n(t) &= k_uP53_n|MDM_{pn}(t) + k_{d_1}P53^u_n|MDM_{pn}(t) + k_{d_u}P53^{uu}_n(t) + c_0P53^u_{pn}(t)\\ &- a_0P53^u_n(t) - k_{a_1}MDM_{pn}(t)P53^u_n(t) - d_4P53^u_n(t) - k_{d_u}P53^u_n(t). \quad (9)\end{aligned}$$

Mono-ubiquitinated phospho-p53. Mdm2-phospho-p53 complex dissociation with ubiquitination + mono-ubiquitinated phospho-p53 with nuclear phospho-Mdm2 complex dissociation without ubiquitination + deubiquitination of double-ubiquitinated phospho-p53 + phosphorylation – dephosphorylation – mono-ubiquitinated phospho-p53 with nuclear phospho-Mdm2 complex formation – degradation – deubiquitination:

$$\begin{aligned}\frac{d}{dt}P53^u_{pn}(t) &= k_uP53_{pn}|MDM_{pn}(t) + k_{d_1}P53^u_{pn}|MDM2_{pn}(t) + k_{d_u}P53^{uu}_{pn}(t) + a_0P53^u_n(t)\\ &- c_0P53^u_{pn}(t) - k_{a_2}MDM_{pn}(t)P53^u_{pn}(t) - d_4P53^u_{pn}(t) - k_{d_u}P53^u_{pn}(t). \quad (10)\end{aligned}$$

Double-ubiquitinated p53. Mono-ubiquitinated p53 with nuclear phospho-Mdm2 complex dissociation with second ubiquitination + dephosphorylation – phosphorylation – effective degradation (summary of downstream dynamics of p53 poliubiquitination and degradation, see main text for further explanation) – deubiquitination:

$$\frac{d}{dt}P53^{uu}_n(t) = k_uP53^u_n|MDM_{pn}(t) + c_0P53^{uu}_{pn}(t) - a_0P53^{uu}_n(t) - d_5P53^{uu}_n(t) - k_{d_u}P53^{uu}_n(t). \quad (11)$$

Double-ubiquitinated phospho-p53. Mono-ubiquitinated phospho-p53 with nuclear phospho-Mdm2 complex dissociation with second ubiquitination + phosphorylation – dephosphorylation – effective degradation (summary of downstream dynamics of p53 poliubiquitination and degradation, see main text for further explanation) – deubiquitination:

$$\frac{d}{dt}P53^{uu}_{pn}(t) = k_uP53^u_{pn}|MDM_{pn}(t) + a_0P53^{uu}_n(t) - c_0P53^{uu}_{pn}(t) - d_5P53^{uu}_{pn}(t) - k_{d_u}P53^{uu}_{pn}(t). \quad (12)$$

Cytoplasmic Mdm2. Translation + dephosphorylation + activation by Nutlin dissociation – phosphorylation (dependent on AKT_p) – degradation – inactivation by Nutlin binding:

$$\begin{aligned}\frac{d}{dt}MDM(t) &= t_0MDM_{RNA}(t) + c_1MDM_p(t) + k_{d_3}MDM_i(t)\\ &- a_1MDM(t)AKT_p(t) - d_0MDM(t) - k_{a_3}MDM(t)NUT(t). \quad (13)\end{aligned}$$

Cytoplasmic phospho-Mdm2. Phosphorylation (dependent on AKT_p)+ import from nucleus + activation by Nutlin dissociation – dephosphorylation – export to nucleus – degradation – inactivation by Nutlin binding:

$$\begin{aligned}\frac{d}{dt}MDM_p(t) &= a_1MDM(t)AKT_p(t) + e_0MDM_{pn}(t) + k_{d_3}MDM_{pi}(t)\\ &- c_1MDM_p(t) - i_0MDM_p(t) - d_0MDM_p(t) - k_{a_3}MDM_p(t)NUT(t). \quad (14)\end{aligned}$$

Nuclear phospho-Mdm2. Nuclear import + activation by Nutlin dissociation – degradation – nuclear export – deactivation by Nutlin binding – complex formation with p53 or phospho-p53 – complex formation with mono-ubiquitinated p53 and phospho-p53 + complex with p53 or phospho-p53 dissociation (with or without ubiquitination) + complex with mono-ubiquitinated p53 or mono-ubiquitinated

phospho-p53 dissociation (with or without ubiquitination).

$$\begin{aligned}\frac{d}{dt}MDM_{pn}(t) = {} & i_0 MDM_p(t) + k_{d_3} MDM_{pni}(t) \\ & - d_0 MDM_{pn}(t) - e_0 MDM_{pn}(t) - k_{a_3} NUT(t) MDM_{pn}(t) \\ & - k_{a_1} MDM_{pn}(t) P53_n(t) - k_{a_2} MDM_{pn}(t) P53_{pn}(t) \\ & - k_{a_1} MDM_{pn}(t) P53^u_n(t) - k_{a_2} MDM_{pn}(t) P53^u_{pn}(t) \\ & + (k_u + k_{d_1}) P53_n|MDM_{pn}(t) + (k_u + k_{d_1}) P53_{pn}|MDM_{pn}(t) \\ & + (k_u + k_{d_1}) P53^u_n|MDM_{pn}(t) + (k_u + k_{d_1}) P53^u_{pn}|MDM_{pn}(t).\end{aligned} \tag{15}$$

Equations of the PTEN-PIP3-Akt module

PTEN transcript. Transcription – degradation:

$$\frac{d}{dt}PTEN_{RNA}(t) = s_1 G_{PTEN}(t) - d_7 PTEN_{RNA}(t). \tag{16}$$

Cytoplasmatic PTEN. Translation – degradation:

$$\frac{d}{dt}PTEN(t) = t_1 PTEN_{RNA}(t) - d_1 PTEN(t). \tag{17}$$

Active PIP3. Activation – deactivation:

$$\frac{d}{dt}PIP_p(t) = a_2(PIP_{tot} - PIP_p(t)) - c_2 PTEN(t) PIP_p(t). \tag{18}$$

Active Akt. Activation – deactivation:

$$\frac{d}{dt}AKT_p(t) = a_3(AKT_{tot} - AKT_p(t)) PIP_p(t) - c_3 AKT_p(t). \tag{19}$$
