# Supplementary material for: The Pharmacodynamics of the p53-Mdm2 Targeting Drug Nutlin: The Role of Gene-Switching Noise
Source: PLoS Comput Biol. 2014 Dec 11;10(12):e1003991. doi: 10.1371/journal.pcbi.1003991 (PMC4263360; doi:10.1371/journal.pcbi.1003991)
Supplement: S2 Text — Hybrid stochastic algorithm and its implementation. (PDF) [file pcbi.1003991.s002.pdf]

Supporting Information file S2

October 28, 2014

S2. Hybrid stochastic algorithm and its implementation

In order to simulate the percentage of viable cells after treatment we performed the stochastic simulation of the response of a sample of cells. As mentioned in the description of the model, we used an hybrid model as well as an hybrid simulation algorithm. In line with the Haseltine and Rawlings algorithm [1], instead of using a full - but computationally very heavy - Gillespie stochastic model, we divided the bioprocesses in two subsets: fast-deterministic and slow-stochastic reactions. In our hybrid model slow reactions are the reactions of gene activation/deactivation, because there are only few copies of each gene. The remaining reactions are considered "fast". Indeed, according to the mass action law, the reaction rate is proportional to the number of molecules, which means that for "fast" reactions the time to the next reaction (at the single molecule level) is actually very small, allowing employing a deterministic approximation. Of course, slow reactions are reactions with small numbers of reactant molecules, where the time between two consecutive molecular reactions is sufficiently long. These reactions require stochastic modeling. In our model there are 6 different slow reactions:

- R1: activation of a Mdm2 gene copy,
- R2: deactivation of a Mdm2 gene copy,
- R3: activation of a PTEN gene copy,
- R4: deactivation of a PTEN gene copy,
- R5: activation of a p53 gene copy,
- R6: deactivation of a p53 gene copy,

and the corresponding propensities are the following:

$$r_1(t) = q^a_{MDM_0} + q^a_{MDM_1} P53^2_{pn}(t) \tag{1}$$

$$r_2(t) = q^d_{MDM} \tag{2}$$

$$r_3(t) = q^a_{PTEN_0} + q^a_{PTEN_1} P53^2_{pn}(t) \tag{3}$$

$$r_4(t) = q^d_{PTEN} \tag{4}$$

$$r_5(t) = q^a_{p53_0} \tag{5}$$

$$r_6(t) = q^d_{p53} \tag{6}$$

The implemented numerical scheme follows the following steps:

1. At the simulation time t, for the given gene states we calculate the total propensity function $r(t)$ of the occurrence of any activation and deactivation reaction:

$$r(t) = r_1(t)(n_{MDM} - G_{MDM}(t)) + r_2(t)G_{MDM}(t) + r_3(t)(n_{PTEN} - G_{PTEN}(t)) + r_4(t)G_{PTEN}(t) + r_5(t)(n_{p53} - G_{p53}(t)) + r_6(t)G_{p53}(t). \tag{7}$$

where n_i denotes total number of "i-th species" alleles and $G_i(t)$ "i-th species" gene state at time t.

2. We select two random numbers p_1 and p_2 from the uniform distribution on $[0, 1]$.
3. Using the fourth order Runge-Kutta solver we evaluate the system of ODEs, accounting for fast reactions, until the time $t + \tau$ such that

$$\ln(p_1) + \int_t^{t+\tau} r(s)ds = 0. \tag{8}$$

In this step we determine which one of the above six possible slow reactions occurs at time $t+\tau$ using the inequality

$$\sum_{i=1}^{k-1} r_i(t + \tau) < p_2 r(t + \tau) \leq \sum_{i=1}^{k} r_i(t + \tau) \tag{9}$$

where k is the index of the reaction to occur.

4. Finally time $t + \tau$ replaces t, and we go back to (1).

Between two consecutive reaction "firings", the ODE system (with constant values for the gene state variables) is regularly sampled each $\Delta t = 0.1$sec to determine if the stochastic reaction occurs in the period time from the last check-time to current check-time. In this method, of course, some inaccuracies may result from the sampling time, although they should be rather rare since gene switching times are in the order of minutes, i.e. much larger than the sampling time Δt.

References

[1] Haseltine HL, Rawlings JB (2002) Approximate simulation of coupled fast and slow reactions for stochastic chemical kinetics. J Chem Phys 117: 6959–6969.
