# Supplementary material for: The Pharmacodynamics of the p53-Mdm2 Targeting Drug Nutlin: The Role of Gene-Switching Noise
Source: PLoS Comput Biol. 2014 Dec 11;10(12):e1003991. doi: 10.1371/journal.pcbi.1003991 (PMC4263360; doi:10.1371/journal.pcbi.1003991)
Supplement: S3 Text — Parameters and their justifications. The rationale of parameter choice is provided. Parameter values are given in Tables 1, 2, and 3. Table 1 reports values taken from the literature, Table 2 values heuristically chosen to fit in vitro dose-response data from Vassilev et al. [6], Table 3 values of parameters characterizing Nutlin pharmacokinetics in mice. (PDF) [file pcbi.1003991.s003.pdf]

Supporting Information file S3

October 30, 2014

S3. Parameters and their justification

The meaning and the values of the parameters we used are reported in Tables 1 and 2. In Table 1 we report the parameter values that were available in experimental literature, with their published range of variation. We give below some details on the rationale for choosing the parameter values listed in the table. On the contrary, in Table 2 we report the parameters for which no experimental data are available (at the best of our knowledge), and for which we found heuristically values able to reasonably fit experimental data.

Parameters of transcription and translation. In the proposed model we adopt the transcription and translation upper limits given by Levin in [1]. We assume that when an allele is active then the transcription proceeds with a constant rate. The speed of mRNA polymerase, ~40 nt/sec, and the characteristic minimum spacing between the neighboring polymerases, 250 nt, give the upper limit for the transcriptional rate of a single allele, $s_{max} = 40/250 = 0.16$ (mRNA/sec). Similarly, we may obtain the upper limit for translation efficiency, $t_{max} = 0.5$ (protein/mRNA/sec). The values of the mRNAs degradation coefficients are taken from the half-lives reported by Sharova [2] for Mdm2, Yang [3] for PTEN and Mazan-Mamczarz [4] for p53. Taking into account the above restrictions, and since Bengtsson et al. have shown that after gene activation the number of mRNA molecules in the single cell can reach $3 \cdot 10^4$ [5], we choose Mdm2, PTEN and p53 gene activation, inactivation, transcription and translation coefficients so that the number of p53 and Mdm2 proteins at the steady state follow the results of Wang et al. [6], i.e. $17 - 37 \cdot 10^3$ p53 molecules and $97 - 221 \cdot 10^3$ Mdm2 molecules.

Parameters involved in the negative feedback loop. In 2006 Tang et al. reported that mutated p53 that cannot create complexes with Mdm2 has half-life time equal approximately 10 hours [7]. Because phosphorylation leads to p53 protection from ubiquitination but not from ubiquitination independent degradation we use the reported half-life time to set the standard degradation rate for both p53 and phospho-p53 to this same value. To determine single and double ubiquitinated p53 degradation rates we used the half-life time reported by Barboza et al. [8] who showed that in the presence of Mdm2 p53 half-life is aproximately equal 15 minutes. Additionally we assumed that the degradation rate for double ubiquitinated p53 is 100 times higher than for single ubiquitinated. For p53-Mdm2 complexes creation and dissolution without ubiquitination rates we used the estimates given by Schon et al. in [9] with the assumption that for phosphorylated p53 the association constant is 10 times smaller than for not phosphorylated. Complexes dissolution with p53 ubiquitination rate is taken from Lai et al. work [10]. Mdm2 half-life without DNA damage was reported by Stommel et al. [11] and Peng et al. [12] to be around 30 minutes.

Parameters involved in the positive feedback loop. The total amount of PIP molecules was taken from Gray et al. work [13] while total amount of Akt from Atrih et al. [14]. Cytoplasmic PTEN degradation rate is taken from Yang et al. work [3] who reported that PTEN half-life is around 10 hours.

References

[1] Levin B (2000) Genes, Vol. VII. Oxford: Oxford University Press.

[2] Sharova LV, Sharov AA, Nedorezov T, Piao Y, Shaik N et al. (2009) Database for mRNA half-life of 19977 genes obtained by DNA microarray analysis of pluripotent and differentiating mouse embryonic stem cells. DNA Res 16: 45–58.

[3] Yang Y, Zhou F, Fang Z, Wang L, Li Z et al. (2009) Post-transcriptional and post-translational regulation of PTEN by transforming growth factor-beta1. J Cell Biochem 106: 1102–1112.

[4] Mazan-Mamczarz K, Galban S, Lopez de Silanes I, Martindale JL, Atasoy U et al. (2003) RNA-binding protein HuR enhances p53 translation in response to ultraviolet light irradiation. Proc Natl Acad Sci USA 100: 8354–8359.

[5] Bengtsson M, Hemberg M, Rorsman P, Stahlberg A(2008) Quantification of mRNA in single cells and modelling of RT-qPCR induced noise. BMC Mol Biol 9: 63.

[6] Wang YV, Wade M, Wong E, Li YC, Rodewald LW et al. (2007) Quantitative analyses reveal the importance of regulated Hdmx degradation for p53 activation. Proc Natl Acad Sci USA 104: 12365–12370.

[7] Tang M, Wahl GM, Nister M (2006) Explaining the biological activity of transactivation-deficient p53 variants. Nat Genet 38: 395–396.

[8] Barboza JA, Iwakuma T, Terzian T, El-Naggar AK, Lozano G (2008) Mdm2 and Mdm4 loss regulates distinct p53 activities. Mol Cancer Res 6: 947–954.

[9] Schon O, Friedler A, Bycroft M, Freund SM, Fersht AR (2002) Molecular mechanism of the interaction between MDM2 and p53. J Mol Biol 323: 491–501.

[10] Lai Z, Ferry KV, Diamond MA, Wee KE, Kim YB et al. (2001) Human mdm2 mediates multiple mono-ubiquitination of p53 by a mechanism requiring enzyme isomerization. J Biol Chem 276: 31357–31367.

[11] Stommel JM, Wahl GM (2004) Accelerated MDM2 auto-degradation induced by DNA-damage kinases is required for p53 activation. EMBO J 23: 1547–1556.

[12] Peng Y, Chen L, Li C, Lu W, Agrawal S et al. (2001) Stabilization of the MDM2 oncoprotein by mutant p53. J Biol Chem 276: 6874–6878.

[13] Gray A, Olsson H, Batty IH, Priganica L, Peter Downes C (2003) Nonradioactive methods for the assay of phosphoinositide 3-kinases and phosphoinositide phosphatases and selective detection of signaling lipids in cell and tissue extracts. Anal Biochem 313: 234–245.

[14] Atrih A, Turnock D, Sellar G, Thompson A, Feuerstein G et al. (2010) Stoichiometric quantification of Akt phosphorylation using LC-MS/MS. J Proteome Res 9: 743–751.

[15] Pan Y, Haines DS (1999) The pathway regulating MDM2 protein degradation can be altered in human leukemic cells. Cancer Res 59: 2064–2067.

[16] Tovar C, Rosinski J, Filipovic Z, Higgins B, Kolinsky K et al. (2006) Small-molecule MDM2 antagonists reveal aberrant p53 signaling in cancer: implications for therapy. Proc Natl Acad Sci USA 103: 10660–10665.

[17] Vassilev LT, Vu BT, Graves B, Carvajal D, Podlaski F et al. (2004) In vivo activation of the p53 pathway by small-molecule antagonists of MDM2. Science 303: 844–848.

[18] Puszynski K, Hat B, Lipniacki T (2008) Oscillations and bistability in the stochastic model of p53 regulation. J Theor Biol 254: 452–465.

[19] Zhang F, Tagen M, Throm S, Mallari J, Miller L et al. (2011) Whole-body physiologically based pharmacokinetic model for Nutlin-3a in mice after intravenous and oral administration. Drug Metab Disp 39: 15–21.

Tables

Table 1: Model parameters: values taken from the experimental literature. All the values are in sec^{-1}.

Name	Meaning	Value	Value range reported in the literature	Source
k_{a_1}	p53–Mdm2 association rate	$8.3 \cdot 10^{-6}$	$(6.5 - 18.4) \cdot 10^{-6}$	[9]
k_{a_2}	phospho-p53–Mdm2 association rate	$8.3 \cdot 10^{-7}$	$(6.5 - 18.4) \cdot 10^{-7}$	[9]
k_{d_1}	dissociation rate of p53–Mdm2 complexes	2	$1.19 - 19.86$	[9]
k_u	p53 ubiquitination rate	0.08	$0.043 - 0.116$	[10]
d_0	Mdm2 degradation rate	$3.85 \cdot 10^{-4}$	-	[11], [12], [15]
d_1	PTEN degradation rate	$1.93 \cdot 10^{-5}$	-	[3]
d_2	p53 degradation rate	$1.93 \cdot 10^{-5}$	-	[7]
d_3	phosphorylated p53 degradation rate	$1.93 \cdot 10^{-5}$	-	[7]
d_4	single-ubiquitinated p53 degradation rate	$1.93 \cdot 10^{-4}$	-	[8]
d_5	double-ubiquitinated p53 degradation rate*	$1.93 \cdot 10^{-2}$	-	[8]
d_6	Mdm2 transcript degradation rate	$6.15 \cdot 10^{-5}$	$(4.23 - 6.15) \cdot 10^{-5}$	[2]
d_7	PTEN transcript degradation rate	$4.81 \cdot 10^{-5}$	$(4.24 - 7.83) \cdot 10^{-5}$	[2], [3]
d_8	p53 transcript degradation rate	$2.13 \cdot 10^{-5}$	$(1.75 - 2.57) \cdot 10^{-5}$	[4]
PIP_{tot}	total amount of PIP molecules	$8 \cdot 10^5$	$(4.8 - 9.1) \cdot 10^5$	[13]
AKT_{tot}	total amount of Akt molecules	$3.4 \cdot 10^4$	$(3.21 - 3.43) \cdot 10^4$	[14]
s_0	Mdm2 mRNA synthesis rate	0.1	$0 - 0.16$	[1]
s_1	PTEN mRNA synthesis rate	0.1	$0 - 0.16$	[1]
s_2	p53 mRNA synthesis rate	0.1	$0 - 0.16$	[1]
t_0	Mdm2 translation rate	0.15	$0 - 0.5$	[1]
t_1	PTEN translation rate	0.005	$0 - 0.5$	[1]
t_2	p53 translation rate	0.03	$0 - 0.5$	[1]
n_{PTEN}	number of PTEN gene copies	2	−	assumed
n_{p53}	number of p53 gene copies	2	−	assumed
n_{MDM}	number of Mdm2 gene copies in RKO cells	2	−	[16]
	in SJSA-1 cells	50	−	[16]

*effective degradation (summary of downstream dynamics of p53 poliubiquitination and degradation, see main text for further explanation)

Table 2: Model parameters: values heuristically chosen to fit dose-response data from [17]. [a], values assumed in [18]; [b], value within the range in [18]. All the values are in sec^{-1} if not differently indicated.

Name	Meaning	Value
k_{a_3}	Nutlin–Mdm2 association rate	$6 \cdot 10^{-5}$
k_{d_3}	Nutlin–Mdm2 dissociation rate	$20 \cdot 10^{-2}$
i_1	rate of Nutlin intracellular import	$1.27 \cdot 10^{8}$ molec/(sec·M)
e_1	rate of Nutlin cell export	$5 \cdot 10^{-3}$
k_{du}	deubiquitination rate of ubiquitinated p53	0.03
a_0	nuclear p53 phosphorylation rate	$1 \cdot 10^{-4}$ [a]
a_1	cytoplasmic Mdm2 phosphorylation rate	$22.5 \cdot 10^{-8}$
a_2	cytoplasmic PIP3 activation rate	$5 \cdot 10^{-5}$ [a]
a_3	cytoplasmic AKT activation rate	$2 \cdot 10^{-9}$ [a]
c_0	nuclear p53 dephosphorylation rate	$8 \cdot 10^{-5}$ [b]
c_1	cytoplasmic MDM2 dephosphorylation rate	$1 \cdot 10^{-4}$ [a]
c_2	cytoplasmic PIP3 deactivation rate (by PTEN)	$2 \cdot 10^{-9}$ [b]
c_3	cytoplasmic AKT deactivation rate	$1 \cdot 10^{-2}$ [b]
i_0	phospho-Mdm2 nuclear import rate	$5 \cdot 10^{-4}$ [a]
e_0	phospho-MDM2 nuclear export rate	0 [a]
$q^a_{MDM_0}$	spontaneous Mdm2 gene activation rate	$2 \cdot 10^{-5}$ [b]
$q^a_{PTEN_0}$	spontaneous PTEN gene activation rate	$2 \cdot 10^{-5}$ [b]
$q^a_{p53_0}$	p53 gene activation rate	$3 \cdot 10^{-5}$
$q^a_{MDM_1}$	p53 driven activation rate of MDM2 gene	$10 \cdot 10^{-13}$ [b]
$q^a_{PTEN_1}$	p53 driven activation rate of PTEN gene	$10 \cdot 10^{-13}$ [b]
q^d_{MDM}	Mdm2 gene deactivation rate	$4 \cdot 10^{-3}$ [b]
q^d_{PTEN}	PTEN gene deactivation rate	$4 \cdot 10^{-3}$ [b]
q^d_{p53}	p53 gene deactivation rate	$8 \cdot 10^{-4}$

Table 3: Parameters of Nutlin binding to plasma and medium proteins, and of Nutlin pharmacokinetics

Name	Meaning		Value	Source
B_{max}	concentration of protein binding sites	in medium	$18 \cdot 10^{-6}$ M	Estimated from [19]
		in plasma	$286 \cdot 10^{-6}$ M	[19]
K_a	equlibrium association constant	in medium	$0.3 \cdot 10^{6}$ M^{-1}	Estimated from [19]
		in plasma	$0.085 \cdot 10^{6}$ M^{-1}	[19]
p_{oral}	dose conversion factor for oral delivery		$0.75 \cdot 10^{-8}$ M/(mg/Kg)	Estimated from [19]
δ_1	gastro-enteric release rate constant		$2 \cdot 10^{-4}$ sec^{-1}	Estimated from [19]
δ_2	elimination rate constant		$5.4 \cdot 10^{-3}$ sec^{-1}	Estimated from [19]

Figures

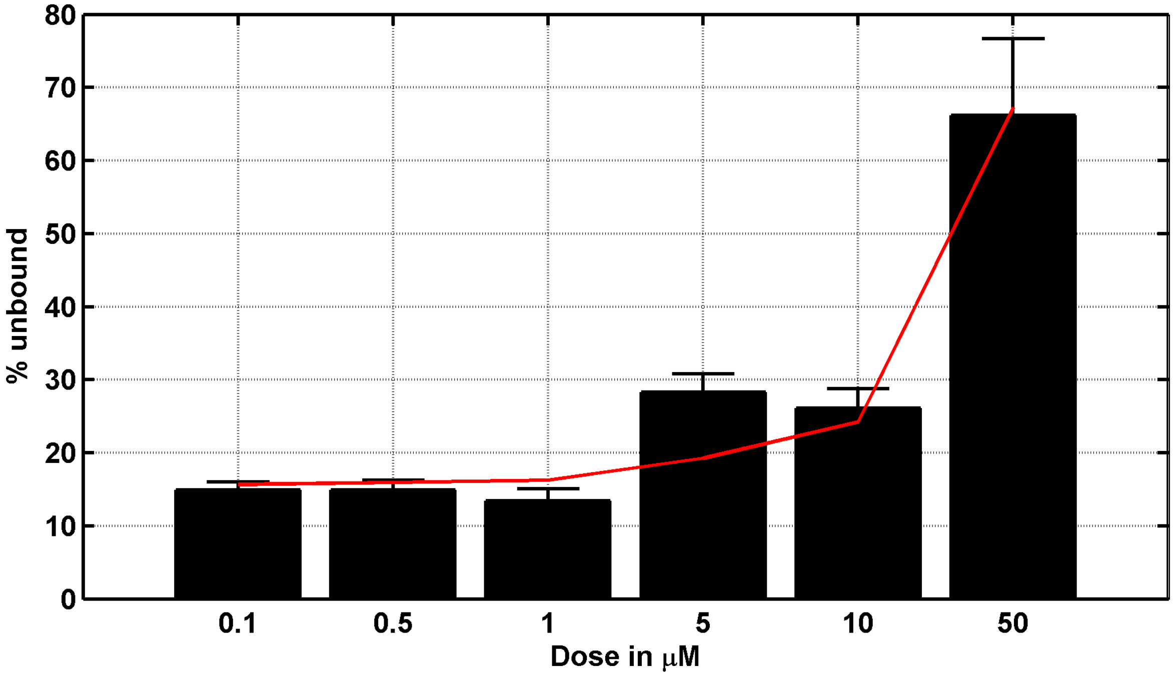

Figure 1: Percentage of unbound Nutlin in the cell culture medium: blacks bars. Red line, fitting by Eq. (6) in main file. Data taken from [19]. Fitting was performed by the least squares method.
