# Supplementary material for: The Pharmacodynamics of the p53-Mdm2 Targeting Drug Nutlin: The Role of Gene-Switching Noise
Source: PLoS Comput Biol. 2014 Dec 11;10(12):e1003991. doi: 10.1371/journal.pcbi.1003991 (PMC4263360; doi:10.1371/journal.pcbi.1003991)
Supplement: S4 Text — Effect of recovery of proliferation after Nutlin-induced cell cycle arrest. (PDF) [file pcbi.1003991.s004.pdf]

Supporting Information file S4

October 28, 2014

S4. Effect of the recovery of proliferation after Nutlin-induced cel cycle arrest

In many cell lines (e.g HCT116 cells [1]), Nutlin preferentially induces only cell cycle arrest, and this cell block may be reversible [2, 3]. To study the effect of the possible recovery of proliferation after Nutlin-induced cell cycle arrest, we implemented in the simulation code a simple algorithm to take into the stochastic duration of the Nutlin-induced rest period.

Let N_{cells} be the number of cells that will be simulated. Let T be the time needed to recover proliferation, which is modeled by means of the following random variable

$$T = T_{min} + \psi,$$

where T_{min} is a deterministic parameter, and ψ is a random variable exponentially distributed with mean ψ_1 whose probability density function is thus given by

$$\varrho(\psi) = \psi_1 e^{(-\psi/\psi_1)}.$$

Note that these assumptions yields:

$$\langle T \rangle = T_{min} + \psi_1.$$

The key steps of the algorithm are the following:

(i) Each single simulation simulates a cell. Each simulation starts at $t = 0$ and ends at $t = t_{end}$; $P53_{pn}(t)$ is monitored during all the length of simulation;

(ii) We define for the *i-th* cell the time-varying binary variable

$$B_i(t) \in \{0, 1\},$$

where 0 stands for non-cycling and 1 for cycling. Of course, for all cells $B_i(0) = 1$;

(iii) We define $N_{viable}(t)$ as

$$N_{viable}(t) = \sum_{1}^{N_{cells}} B_i(t);$$

(iv) If a time $\hat{t}$ exists such that for $t \in (\hat{t}, \hat{t} + \Delta)$, $\Delta = 1\text{h}$, it is $P53_{pn}(t) > \theta$, where θ is a suitable threshold, then at $t = \hat{t} + \Delta$ the cell cyle stops and the cell is considered no more viable. Thus, we set

$$B_i(\hat{t} + \Delta) = 0;$$

(v) Let $t_{drop} > \hat{t} + \Delta$ be the time at which $P53_{pn}$ possibly decreases below the threshold. The random number T is then extracted, and

$$t_{recovery} = t_{drop} + T$$

is computed;

(vi) If the p53 level remains under threshold until $t_{recovery}$, we set

$$B_i(t_{recovery}) = 1;$$

If instead, before $t_{recovery}$, the p53 level crosses again the threshold for a time interval Δ, the new $t_{recovery}$ is determined based on the new t_{drop} so the recovery clock resets itself;

(vii) The above steps are performed for all the simulated cells;

(viii) Let t_{end} be the time at which the viability is evaluated. N_{viable} at t_{end} is computed by:

$$N_{viable}(t_{end}) = \sum_{1}^{N_{cells}} B_i(t_{end}).$$

By means of the above described algorithm, for various values of T_{min} we performed simulations of the dose-response curve experimentally measured in [4], and of the response to oral Nutlin delivery (single dose and split doses delivery, with the same modalities as reported in the main text). The value of the average ψ was set to $\psi = 13\ h$, whereas the values of the other parameters are those of Table 1 and 2 in Supplementary Information S3 with no amplification of Mdm2 gene, i.e. they are the parameters we adopted for RKO cells. These parameters can also describe the behavior of the HCT116 cell line.

In case of oral bolus delivery, it is quite evident the great influence of the reversibility of the cell cycle arrest on the viability assessment when the dose is given as single bolus or split with 6h breaks. In these cases, there is a substantial time interval from the peak (or peaks) of Nutlin concentration and the time of viability assessment (see Figures 7 and 8 of the main text). Apart from the case of the shortest recovery time (T_{min} = 24h), a reduced effect may be observed when the oral delivery is split with 24h breaks, and when the experiment by Vassilev et al. [4] is considered. In both these simulations, indeed, the assessment time is close to the end of the exposure to Nutlin. When it occurs, in fact, the possible under-threshold drop of active p53 triggering the recovery process is a random event that we can roughly considered to be uniformly distributed during the exposure period. Thus only a fraction of these events can lead to the proliferation recovery before the assessment time. Of course, the more this time is delayed with respect to the end of the effective drug exposure, the more the viability will be fully recovered.

References

[1] Tovar C, Rosinski J, Filipovic Z, Higgins B, Kolinsky K et al. (2006) Small-molecule MDM2 antagonists reveal aberrant p53 signaling in cancer: implications for therapy. Proc Natl Acad Sci USA 103: 10660–10665.

[2] Huang B, Deo D, Xia M, Vassilev LT (2009) Pharmacologic p53 activation blocks cell cycle progression but fails to induce senescence in epithelial cancer cells. Mol Cancer Res 7: 1497–1509.

[3] Korotchkina LG, Demidenko ZN, Gudkov AV, Blagosklonny MV (2009) Cellular quiescence caused by the Mdm2 inhibitor nutlin-3A. Cell Cycle 8: 3777–3781.

[4] Vassilev LT, Vu BT, Graves B, Carvajal D, Podlaski F et al. (2004) In vivo activation of the p53 pathway by small-molecule antagonists of MDM2. Science 303: 844–848.

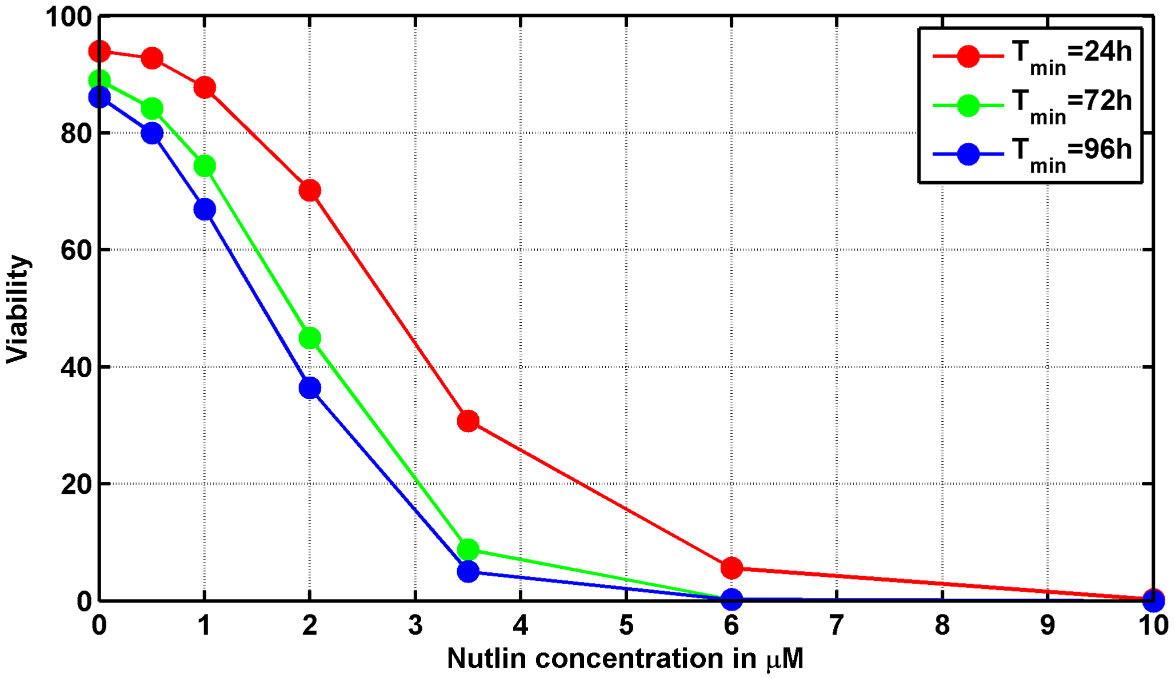

Figure 1: Effect of different proliferation recovery times on the outcome of the experiment by Vassilev et al. [4]. The parameter ψ_1 is equal to 13h. Viability is assessed at 120h from the start of exposure (i.e at the end of the exposure to Nutlin). 250 cells are simulated.

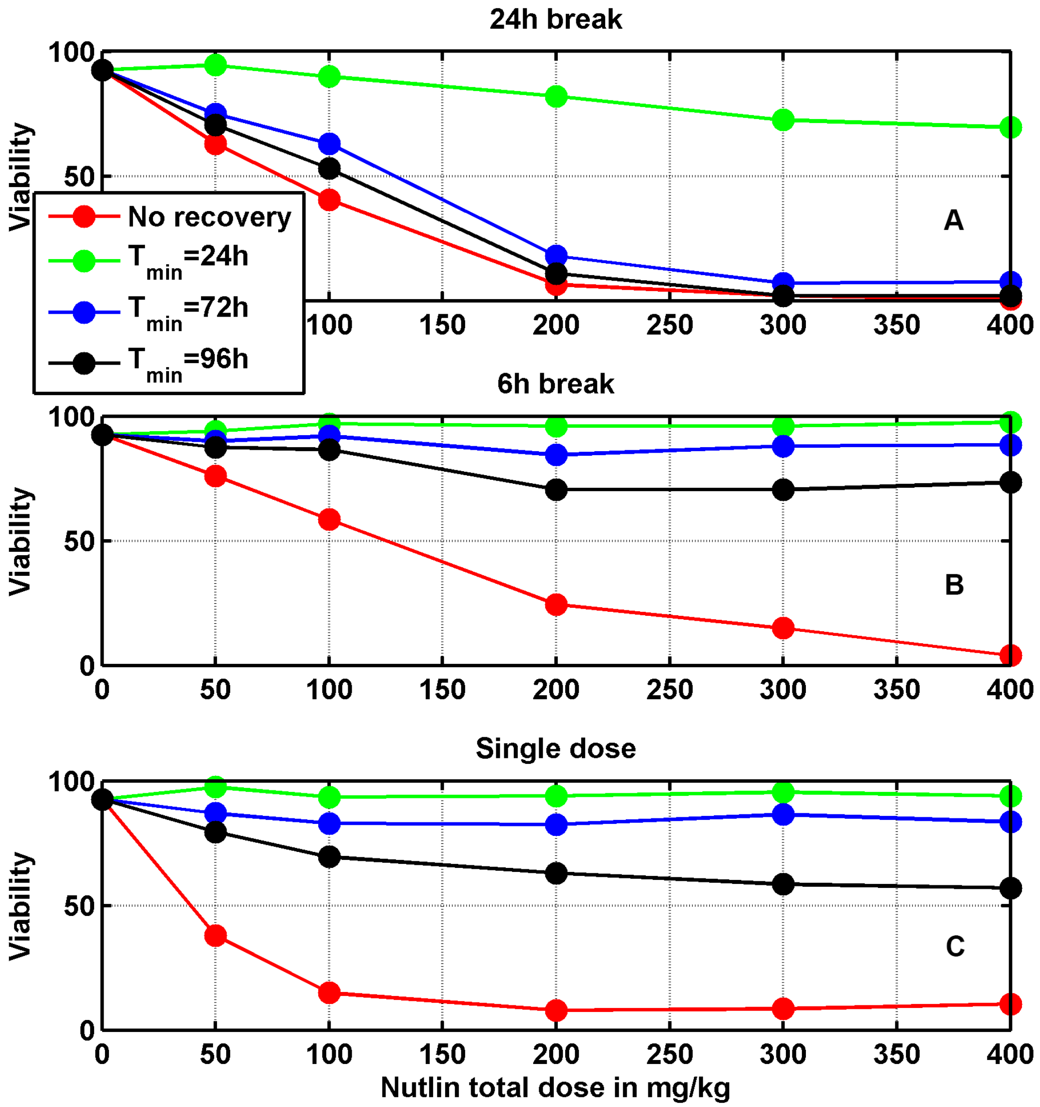

Figure 2: Effect of different proliferation recovery times on the response of oral dose delivery. The parameter ψ_1 is equal to 13h. Viability is assessed at 120h after the first bolus delivery. 250 cells are simulated
